# Towards Covalent Photosensitizer-Polyoxometalate Dyads-Bipyridyl-Functionalized Polyoxometalates and Their Transition Metal Complexes

**DOI:** 10.3390/molecules24244446

**Published:** 2019-12-04

**Authors:** Andreas Winter, Patrick Endres, Erik Schröter, Michael Jäger, Helmar Görls, Christof Neumann, Andrey Turchanin, Ulrich S. Schubert

**Affiliations:** 1 Laboratory of Organic and Macromolecular Chemistry (IOMC), Friedrich Schiller University Jena, Humboldtstr. 10, 07743 Jena, Germany; andreas.winter@uni-jena.de (A.W.); patrick.endres@uni-jena.de (P.E.); erik.schroeter@uni-jena.de (E.S.); michael.jager.iomc@uni-jena.de (M.J.); 2 Center for Energy and Environmental Chemistry (CEEC) Jena, Friedrich Schiller University Jena, Philosophenweg 7a, 07743 Jena, Germany; christof.neumann@uni-jena.de (C.N.); andrey.turchanin@uni-jena.de (A.T.); 3 Institute for Inorganic and Analytical Chemistry (IAAC), Friedrich Schiller University Jena, Humboldtstr. 8, 07743 Jena, Germany; helmar.goerls@uni-jena.de; 4 Institute of Physical Chemistry (ICP), Friedrich Schiller University Jena, Lessingstr. 10, 07743 Jena, Germany

**Keywords:** hybrid materials, metal complexes, polyoxometalates, photocatalysis

## Abstract

A triol-functionalized 2,2′-bipyridine (bpy) derivative has been synthesized and used for the tris-alkoxylation of polyoxometalate (POM) precursors. The resultant POM-bpy conjugates of the Wells–Dawson- and Anderson-type feature a C–C bond as a linkage between the POM and bpy fragments. This structural motif is expected to increase the hydrolytic stability of the compounds. This is of particular relevance with respect to the application of POM-bpy metal complexes, as photocatalysts, in the hydrogen-evolution reaction (HER) in an aqueous environment. Accordingly, Rh(III) and Ir(III) complexes of the POM-bpy ligands have been prepared and characterized. These catalyst-photosensitizer dyads have been analyzed with respect to their electrochemical and photophysical properties. Cyclic and square-wave voltammetry, as well as UV/vis absorption and emission spectroscopy, indicated a negligible electronic interaction of the POM and metal-complex subunits in the ground state. However, emission–quenching experiments suggested an efficient intramolecular electron-transfer process from the photo-excited metal centers to the POM units to account for the non-emissive nature of the dyads (thus, suggesting a strong interaction of the subunits in the excited state). In-depth photophysical investigations, as well as a functional characterization, i.e., the applicability in the HER reaction, are currently ongoing.

## 1. Introduction

The molecular metal oxides, the so-called polyoxometalates (POMs), represent an important class of materials with high relevance for a range of potential applications. Their chemical and electronic properties can be modulated by their chemical composition, and, for example, the pronounced Brønsted acidity can be employed to catalyze a range of organic transformations (e.g., esterification, hydrolysis, etc.) [1]. Moreover, their ability to undergo redox processes in a fast and highly reversible manner enables their usage as a catalyst for the oxidation of inter alia alcohols and sulfides, alkenes or arenes. In addition, the reduction of organic substrates, in particular, the photoreduction of CO_2_, has attracted considerable interest [2]. Even more importantly, POMs have found to be active in the chemical, photochemical, and electrochemical production of O_2_ from water, hence, facing the challenge of converting solar energy into chemical bonds [2,3]. The same also holds true for the photo-/electrochemical reduction of protons to yield H_2_ as a clean and renewable energy carrier. The limitation of this application, the need of hard UV irradiation to activate the POM catalysts, can be overcome by introducing a photosensitizer (PS) into the catalytic system; as a consequence, visible light can be harvested to achieve the photochemical H_2_ evolution [2,4,5]. Organic dyes, as well as transition metal complexes, such as [Ru(bpy)_3_]^2+^ or [Ir(ppy)_2_(bpy)]^+^, have been used in this respect (bpy: 2,2′-bipyridine, ppy: 2-phenylpyridine).

It has been shown that the covalent linkage of organic moieties to polyoxometalates yields hybrid materials with unique properties that can be exploited in the context of bioinorganic hybrids (i.e., POMs as “inorganic amino acids”) [6], (supra)molecular nano-assemblies [7], light-driven H_2_ production [3,8,9,10] or electrochemical energy storage [11,12]. In general, covalent systems can be utilized for the conversion of sunlight into chemical energy [13]. This well-established doctrine also applies to sensitizer-POM conjugates as designated photocatalysts for the light-driven hydrogen evolution reaction (HER) [3]. In pioneering work in this field, the groups of Proust, Izzet, and Artero reported such dyads in which the Ru(II) or Ir(III) sensitizer was attached to redox-active Keggin- or Dawson-type POMs via carbon–silicon bonds. These materials exhibited a significantly better performance in the HER when compared to analogous noncovalent catalyst systems as references [8,14,15,16,17]. Recently, Streb and co-workers observed the same behavior of an increased HER activity as an example of Anderson-type POMs with two pending Ir(III) sensitizers [10,18]. This finding can be attributed to an improved charge separation and electron transfer between the subunits in the covalently bound materials. These issues have been analyzed by the groups of Odobel, Mayer, and Harriman [19], Hanan and Hasenknopf [20], as well as Schubert and Dietzek [21,22].

With respect to the synthesis of sensitizer-POM dyads, various methods can be applied [23]. From these, the tris-alkoxylation of POM precursors by triol-functionalized organic molecules is of particular virtue [24]. Tripodal alcohols, such as TRIS (TRIS: tris(hydroxymethyl)-aminomethane) or its derivates, can be utilized for the organo-functionalization of Anderson-, Lindqvist- or Dawson-type POMs. The vacant amino groups of the TRIS-POM conjugated can be utilized for a subsequent attachment of additional functional molecules, such as Ir(III) photosensitizers, in Schiff-base reactions [18]. Hanan and Hasenknopf introduced an alternative approach in which polypyridyl ligands equipped with a triol anchor group were used to synthesize POMs with pending ligands for transition metal ion complexation [20,25,26]. Recently, aryl-substituted derivatives of tris(hydroxymethyl)methane have been suggested as tripodal ligands for the organo-functionalization of POMs [24]. Yang and co-workers prepared a bis-functionalized Anderson-type POM from 2-(hydroxymethyl)-2-(pyridin-4-yl)propane-1,3-diol as an alkoxylation substrate; the POM hybrid was assembled further into a heterometallic cluster-organic framework with cuprous iodide [27]. A range of solid-state coordination polymers was prepared from the same POM hybrid with the divalent cations Cu(II) and Zn(II) [28]. Nachtigall et al. applied a solvothermal reaction protocol of V(O)(SO_4_) to incorporate the pyridyl building block into functionalized POM fragments [29].

In the present work, we report the synthesis of new bpy-functionalized Anderson- and Dawson-type POMs, along with their phenyl-carrying counterparts, and their utilization to complex Rh(III) and Ir(III) centers. In contrast to the aforementioned examples known from the literature, the POM–photosensitizer conjugates presented herein feature a covalent C–C bond as the covalent linkage between the subunits. This connection is expected to be beneficial in two ways: First, the rigid and short connection will facilitate a strong and vectoral excited-state coupling between the metal complex and the POM acceptor. Second, the chemical robustness will increase the stability against hydrolytic decomposition of the dyads when applied as photocatalysts in the HER, in particular in aqueous environments, such as DMF/water mixtures. Herein, the synthesis and structural characterization of the compounds is presented, while the detailed spectroscopic and functional characterization with respect to an application in the HER is subject to ongoing research.

## 2. Results and Discussion

### 2.1. Synthesis and Characterization of the Organo-Functionalized POMs

**Scheme 1:**
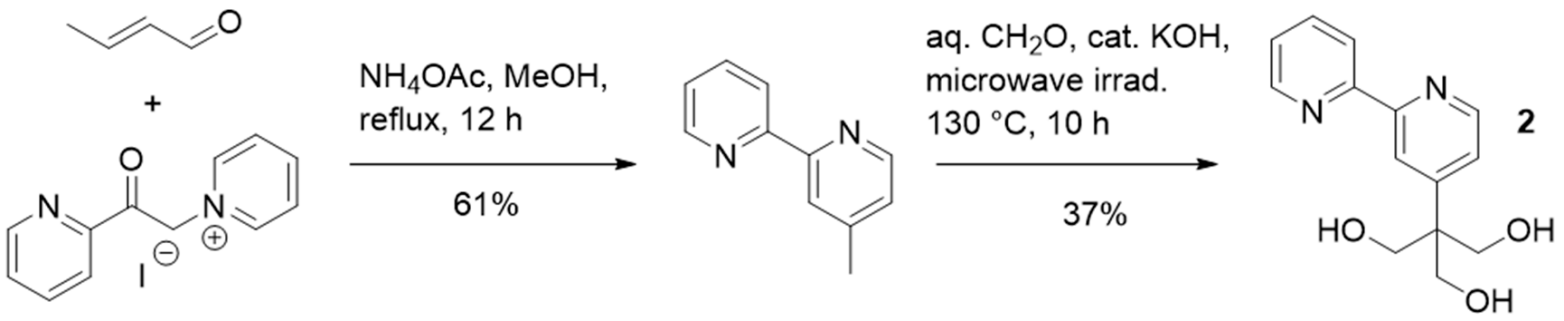
Schematic representation of the two-step synthesis of 2,2′-bipyridine (bpy)-triol **2**.

To prepare the functionalized polyoxometalates **3**–**6** via the tris-alkoxylation method, the corresponding triols **1** and **2** had to be synthesized first. Following a literature procedure, **1** was obtained from phenylacetaldehyde in a Tollens-type condensation in a 37% yield [30,31]. The two-step synthesis of bpy-triol **2** is outlined in Scheme 1. The Kröhnke-type condensation of crotonaldehyde afforded 4-methyl-2,2′-bipyridine in a 61% yield [32]. This yield is much higher than reported in Constable’s original protocol and is a result of the optimized purification procedure: The crude methanolic reaction mixture was extracted continuously with *n*-hexane for several days (the pure product was subsequently isolated from the hexane phase). The base-catalyzed hydroxymethylation of 4-methyl-bpy, inspired by Menozzi’s hydroxymethylation of 4-picoline [33], was performed under highly forcing conditions (microwave irradiation, 130 °C, 10 h). After acidic hydrolysis of the intermediate hemi- and full acetals, **2** was isolated in moderate yield (37%).

**Scheme 2:**
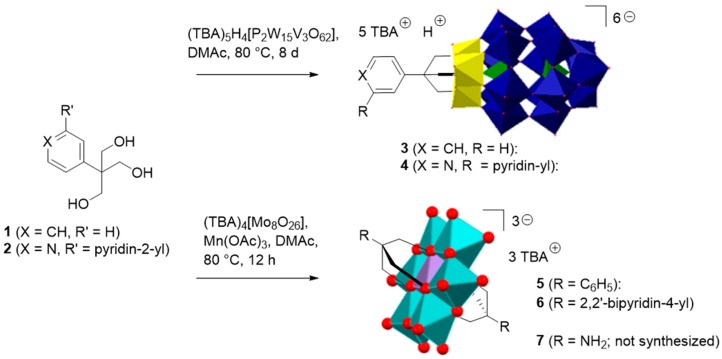
Schematic representation of the synthesis of the phenyl and bpy-equipped POMs **3**–**6**. Color legend for compounds **3** and **4**: V yellow, P green, dark blue W; color legend for compounds **5**–**7**: O red, Mo cyan, Mn violet.

As outlined in Scheme 2, the triols **1** and **2** were reacted with the corresponding POM precursor materials [34,35], i.e., (TBA)_5_H_4_[P_2_W_15_V_3_O_62_] and (TBA)_4_[Mo_8_O_26_] in the presence of Mn(OAc)_3_ (TBA: tetra(*n*-butyl)ammonium) for the Dawson- (**3** and **4**) and Anderson-type materials (**5** and **6**), under the established conditions of POM alkoxylation in *N*,*N*-dimethylacetamide (DMAc) [36,37]. The hybrids were isolated in satisfying yields as their TBA salts with reasonable solubility in polar-aprotic solvents (e.g., DMSO, DMF, acetonitrile). All the functionalized POMs were characterized by NMR spectroscopy and mass spectrometry (all spectra are depicted in the Supplementary Material). In brief, the Dawson-type POMs **3** and **4** showed the expected ratio of integrals between the signals arising from the pending phenyl or bipyridyl moiety, the CH_2_ groups, and the accompanying counter cations in the ^1^H NMR spectrum. Moreover, the characteristic signals in the ^31^P NMR spectrum for the two different P atoms within the {V_3_}-capped cluster framework could also be observed (*δ* ≈ −7.9 ppm and −13.0 ppm) [36]. Due to the presence of the paramagnetic Mn(III) center in the Anderson-type systems **5** and **6**, the signals in the aromatic region of the ^1^H NMR spectra appeared broadened but still could be related to those of the TBA cations. As a distinct feature, the signals of the CH_2_ groups experienced a pronounced paramagnetic downfield shift and could be identified as a broad unstructured signal at ca. 64 ppm [37]. The paramagnetic nature of the Mn(III) center also prevented the probing of **5** and **6** by ^95^Mo NMR spectroscopy—a technique commonly used to analyze Mo-containing POMs (note, that the characteristic signal at 18.0 ppm was observed for the α-[Mo_8_O_26_]^4–^ precursor when applying this method, Figure SM-8) [38].

**Figure 1:**
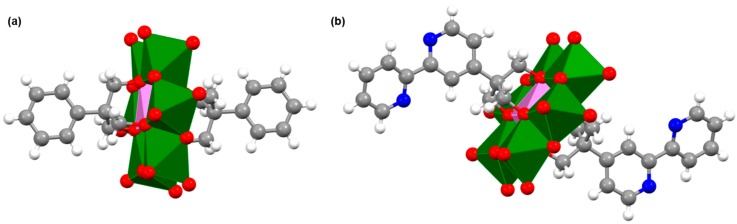
Side view of the solid-state structures of **5** (**a**) and **6** (**b**). Color legend: H white, C grey, N blue, O red, Mo green, Mn violet. The tetra(*n*-butyl)ammonium (TBA) cations and solvent molecules are omitted for clarity, ellipsoids are shown at the 50% probability level.

In the case of the Anderson-type POMs **5** and **6**, single crystals suited for X-ray structure analysis could be obtained by diffusion of diethyl ether into the DMAc solutions. The crystal structure parameters and the refinement details are given below (see the Experimental Section). The phenyl-bearing POM derivative **5** crystallized in a monoclinic space group C2/c with Z = 4, whereas the bpy-bearing derivative **6** yielded an orthorhombic space group Pca2_1_ with Z = 4. As depicted in Figure 1, the typical structure for symmetrically disubstituted Anderson POMs was found (i.e., the so-called *δ*-form with almost *D*_3_ symmetry) [39]. The Mo–O bond distances, as well as the O–Mo–O bond angles, were in the normal dimensions for such clusters [37]. The central Mn(III) ion was surrounded by six O atoms carrying the two tripodal organic ligands, the Mn–O bond lengths were in the range of 1.926(8) to 2.039(8) Å; whereas the C–O bond lengths were in the range from 1.436(5) to 1.442(6) Å.

**Figure 2:**
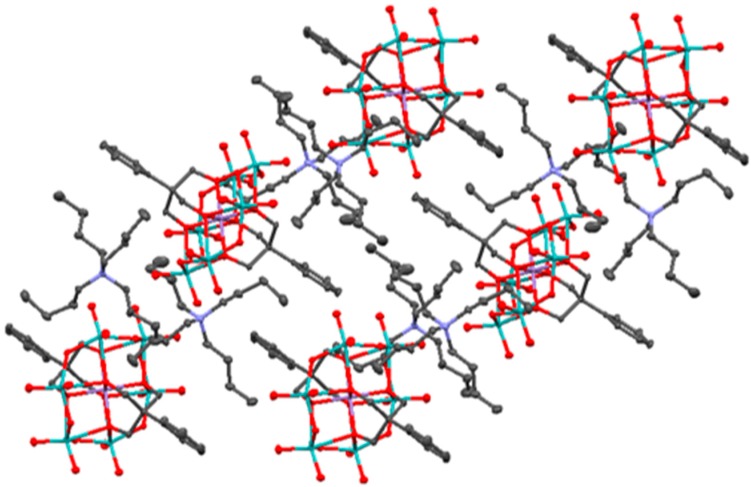
Representation of the molecular packing of the structure of **5**. Color legend: C grey, O red, N blue, Mo green, Mn magenta. The hydrogen atoms and solvent molecules are omitted for clarity.

Due to the more rigid-linear structure arising from the direct connection of the phenyl rings to the tripodal anchor groups, the molecular packing of **5** (Figure 2) is significantly different to that reported for a related POM hybrid where two phenyl moieties have been attached to a bis-tris-functionalized Anderson POM via imine linkages [40]. In the present case, the interplay of different supramolecular interactions results in an overall layer-like arrangement. In detail, chains of **5** are formed in which two TBA cations always separate the polyanions from each other. Thereby, the TBA cations interact with the POM cores via C–H⋅⋅⋅O hydrogen bonding [40]. These intermolecular interactions are mainly formed between the terminal oxo-moieties of the {MnMo_6_} core and various C–H moieties of the TBA cations (Mo–O–Mo fragments appear to be less involved in this). More important, the POM-TBA chains intercalate into each other via ππ-stacking interactions between the almost coplanar aromatic rings. Due to interference by the alkyl chains of the TBA cations, a strictly alternating order of close and more remote ππ-stacks could be observed (i.e., 4.430(4) vs. 7.885(4) Å). For **6**, the packing in the crystalline state occurred in a different fashion (see the Supplementary Material). In particular, the ππ-stacking interactions between the bpy substituents was only expressed weakly. Instead, C–H⋅⋅⋅N hydrogen bonds between the organic residues could be identified. As in the previous case, a chain-like assembly motive of the POM unit was adopted with the bpy moieties tilted by almost 90° with respect to those of the neighboring molecules. Moreover, close interchain hydrogen-bonding interactions involving the terminal oxo-moieties of the {MnMo_6_} core and the unsubstituted pyridine rings contributed to the overall packing mode.

### 2.2. Synthesis and Characterization of the Photosensitizer-Polyoxometalate Dyads

**Scheme 3:**
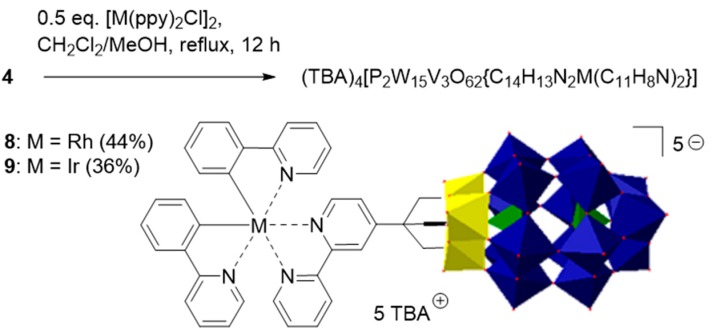
Schematic representation of the synthesis of the photosensitizer-POM dyads **8** and **9**.

**Scheme 4:**
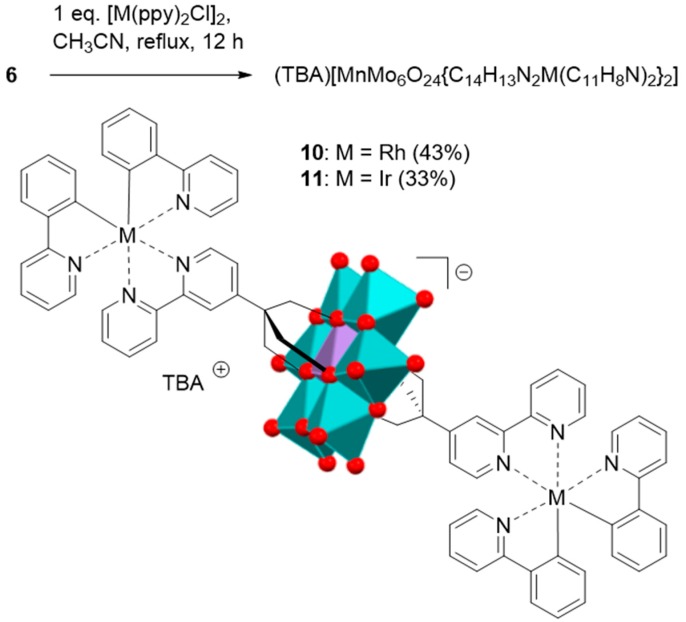
Schematic representation of the synthesis of the photosensitizer-POM dyads **10** and **11**.

As described previously by the groups of Hasenknopf [26] and Streb [10], the bpy-equipped POMs represent versatile platforms for the coordination of transition metal complexes, such as biscyclometalated Rh(III) and Ir(III) or Re(I) tricarbonyl fragments. In line with these studies, the coordination of appropriate precursor complexes to the POM-bpy conjugates was investigated. Accordingly, the reaction of **4** with the μ-dichloro-bridged dimeric [M(ppy)_2_Cl]_2_ precursor complexes (M = Ir or Rh) in dichloromethane/methanol mixtures (5:2 *v*:*v*) yielded the dyads **8** and **9** (Scheme 3). The complexation of the Anderson-type POM-bpy conjugate **6** had to be performed at a higher temperature and, thus, acetonitrile was used as a solvent in these cases. This protocol yielded the dinuclear assemblies **10** and **11** (Scheme 4).

**Figure 3:**
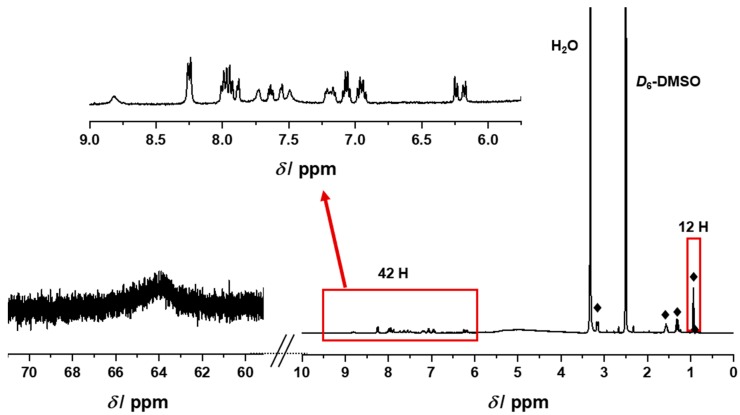
^1^H NMR spectrum of **11** (300 MHz, *D*_6_-DMSO); the low-field region displaying the broad signal of the CH_2_ groups is shown as magnification. The signals of the TBA^+^ are marked (♦).

**Figure 4:**
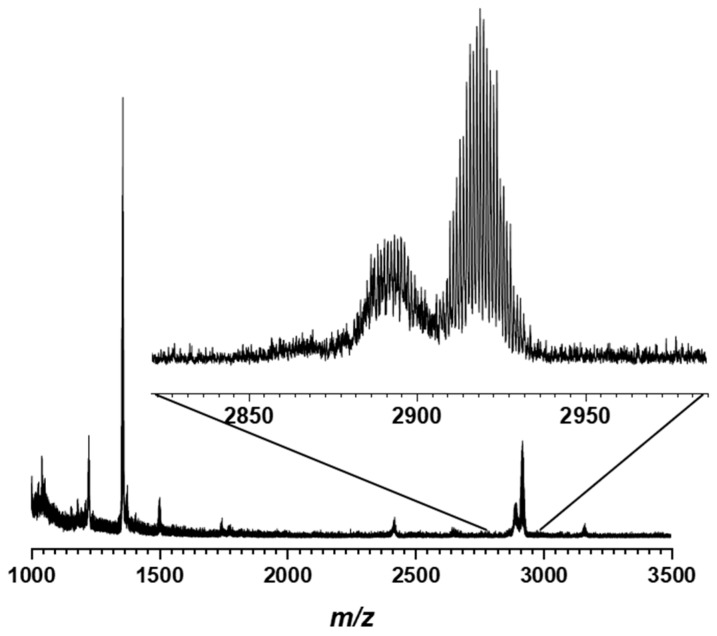
MALDI-TOF mass spectrum of **11** (*trans*-2-[3-(4-*tert*-butylphenyl)-2-methyl-2-propenylidene]malononitrile (commonly abbreviated as DCTB) as matrix, KCl as ionization agent).

The identity and purity of all photosensitizer-POM dyads were concluded from NMR and X-ray photoelectron spectroscopy (XPS) as well as mass spectrometry (see the Supplementary Material). Representatively, the ^1^H NMR and MALDI-TOF spectra of the dinuclear POM-Ir(III) dyad **11** are depicted in Figure 3 and Figure 4, respectively. In the case of the Anderson-type PS-POM dyads **10** and **11**, the characteristic broad signal at ca. 64 ppm, assigned to the CH_2_ groups of the trialkoxy linker, was observed. Single crystals suited for X-ray structure determination could, however, not yet be obtained in any case. The elemental composition of the PS-POM dyads was determined by XPS. The overview spectra, as summarized in Figure SM-26, revealed the expected elements of the dyads **8–11**. A closer inspection of the high-resolution spectra revealed the characteristic signals of the heavy-metal atoms, e.g., the Rh 3d as well as the Ir 4f and Mo 3d signals in the dyads **8** and **11**, respectively (Figures SM-27 and SM-28): The spin-orbit splitting of the d and f photoelectrons gave rise to a signal splitting. The quantitative analysis of these spectra enabled to determine the elemental composition. For example, Figure SM-28 was used to determine the Ir-to-Mo ratio of 1:(3.1 ± 0.2), which was in a very good agreement with the expected 1:3 stoichiometry. For all dyads, the relevant elemental ratios were determined in the same way; the data are summarized in Table 1. The results matched the calculated ratios and, thus, verified the chemical structure of the dyads. In short, the observed W-to-P ratios of (5.9 ± 0.2):1 and (6.4 ± 0.2):1 in the case of **8** and **9**, respectively, confirmed the integrity of the POM subunit after complexation (the theoretical W-to-P ratio is 6:1). In addition, the derived Rh-to-P and Ir-to-P ratios corroborated the successful coordination of the [(ppy)_2_M]^+^ fragments to **4**. The same also holds true for the dyads **10** and **11**: The experimental Mo-to-Mn ratios of (6 ± 0.3):1 fully matched with the theoretical ones; the Rh-to-Mo and Ir-to-Mo ratios [1:(2.9 ± 0.2) and 1:(3.1 ± 0.2)] were also very close of the expected 1:3 ratio. Thus, the twofold complexation of **5** by [(ppy)_2_M]^+^ fragments could be confirmed based on the XPS results. The summary of the analyzed XP spectra and more detailed information about the quantitative analysis are presented in the Supplementary Material (Table SM-1).

### 2.3. Photophysical and Electrochemical Properties of the Dyads Based on the Wells–Dawson-Type POM

#### 2.3.1. Electrochemical Properties

To investigate the electrochemical properties of the materials possessing a Wells–Dawson-type POM, cyclic voltammetry (CV) and square-wave voltammetry (SWV) measurements were performed in dry acetonitrile (containing 0.1 M TBAF as electrolyte) to circumvent the reported adsorption phenomena on the electrode upon reduction when using DMF as a solvent. The observed redox potentials are summarized in Table 2.

The Ph-POM conjugate **3** was used as reference material. Due to the absence of any redox-active organic moiety, all reduction and oxidation processes were expected to occur on the POM fragment. The CV of **3** showed three signals in the regime from 0 to −2 V (Figure SM-29a). From these, only the signal at −1.34 V might be considered as reversible. To gain further insight into the redox properties of **3**, SWV was applied additionally (Figure SM-29b). At positive voltages, three signals were identified (0.69, 0.43, and 0.09 V), which were correlated with oxidation processes involving the {V_3_} tris-vanadium cap of the POM fragment. These results agree with the data reported by Auvray et al. elsewhere for related organo-functionalized Dawson-type POMs [26]. The same also holds true for the four reduction processes which were assigned to the cluster; the first two are expected to occur on the V(V) centers. According to the available literature report, these reductions might be observed either as three individual one-electron or two separated processes, in which a one-electron reduction is followed by a two-electron reduction [41]. The latter is in line with the experimental data. The additional reduction processes involve the rest of the cluster, but no clear assignment can yet be made. Similar signal patterns have been reported in the literature. However, no statement about their exact origin has been made [26,41]. Presumably, the intense signal at −1.34 V is due to a reduction of the tungsten centers.

The electrochemical analysis of the Rh(III)-containing dyad **8** basically yielded the same results as for the model compound **3** (though the CV and SWV were poorly resolved, Figure SM-30). In addition to the oxidation of the {V_3_} cap, the irreversible signal at ca. 1.4 V was assigned to the oxidation of the Rh(III) center. At negative potentials, two prominent signals were found which were assigned to reductions of the bpy and ppy ligands of the attached complex. The multiple-electron reduction of the cluster appeared as a broad, unstructured signal at ca. −1 V. In addition, for the Ir(III)-containing counterpart **9,** an irreversible metal-based oxidation at 1.0 V became apparent in the CV curve (Figure SM-31). The SWV analysis of **9** yielded basically the same features as for **8**, though the overlapping of the signals prevents any detailed discussion.

#### 2.3.2. UV/vis Absorption and Emission Studies

**Figure 5:**
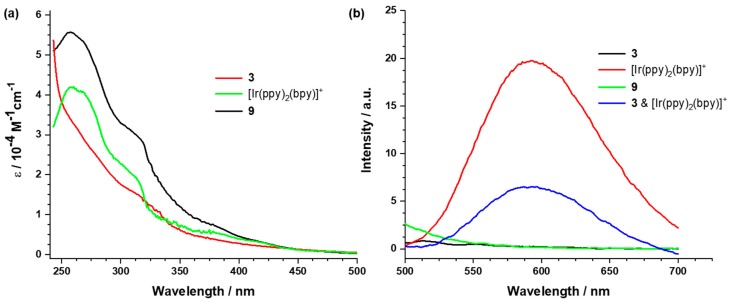
UV/vis absorption (**a**) and emission spectra (**b**) of **3**, [Ir(ppy)_2_(bpy)]^+^ and **9** (all spectra were measured at room temperature in CH_3_CN (c = 10^−5^ mol L^−1^).

The UV/vis absorption spectra of the compounds were recorded in CH_3_CN; the absorption bands and the corresponding molar extinction coefficients are summarized in Table 3. Both compounds **3** and **4** revealed intense absorption of UV light due to π→π* transitions in the organic unit (Figure 5a). These were accompanied by ligand-to-metal charge-transfer (LMCT) bands, which tailed from the UV regime into the high-energy visible light part of the spectrum; the bands were assigned to the characteristic O→W(VI) and O→V(V) transitions of the Wells–Dawson-type POM structure.

The [Ir(ppy)_2_(bpy)]^+^ model complex showed typical absorption bands at 258, 310, and 380 nm, which correspond to π→π*, metal-to-ligand charge-transfer (MLCT) and metal-centered (MC) transitions, respectively [42]. As depicted in Figure 5a, these bands could also be observed for the dyad **9**; the only marginal shift of the bands suggests a negligible electronic interaction between the metal complex and the POM in the ground state. The increased molar absorption of **9** compared to the model complex is due to the contributions of the POM in the UV to the high-energy visible regime. Basically, the same photophysical behavior was found for the Rh(III)-containing dyad **8**, i.e., the characteristic absorption bands known from the [Rh(ppy)_2_(bpy)]^+^ complex (λ_abs_ = 258, 303, and 370 nm) were also present in **8** and electronic ground-state interactions between the subunits were again excluded (Figure SM-35).

The [Ir(ppy)_2_(bpy)]^+^ complex is brightly luminescent at room temperature; upon excitation in its MLCT band, intense emission at 592 nm can be observed [43]. However, the dyad **9** did not exhibit any luminescence. On the other hand, a stoichiometric mixture of **3** and [Ir(ppy)_2_(bpy)]^+^ revealed a residual emission with approximately 25% of the intensity of the small-molecule reference complex (Figure 5b). Detailed photophysical studies on the Ir(III)-containing dyads **9** and **11** with respect to the quenching mechanism are currently ongoing. However, the results line up with the observations already made for related POM-Re dyads, for which intramolecular electron-transfer processes were proposed [26]. Auvray et al. pointed out that the luminescence quenching via such a pathway would be significantly more efficient in molecular dyads comprising a covalent linkage of the components; for the intermolecular electron transfer, as in the mixture of **3** and [Ir(ppy)_2_(bpy)]^+^, the diffusion rate will limit the quenching efficiency. Due to the non-emissive nature of [Rh(ppy)_2_(bpy)]^+^ under the experimental conditions, a similar quenching study could not be performed with compound **8**.

### 2.4. Photophysical and Electrochemical Properties of the Dyads Based on the Anderson-Type POM

#### 2.4.1. DFT Studies

**Figure 6:**
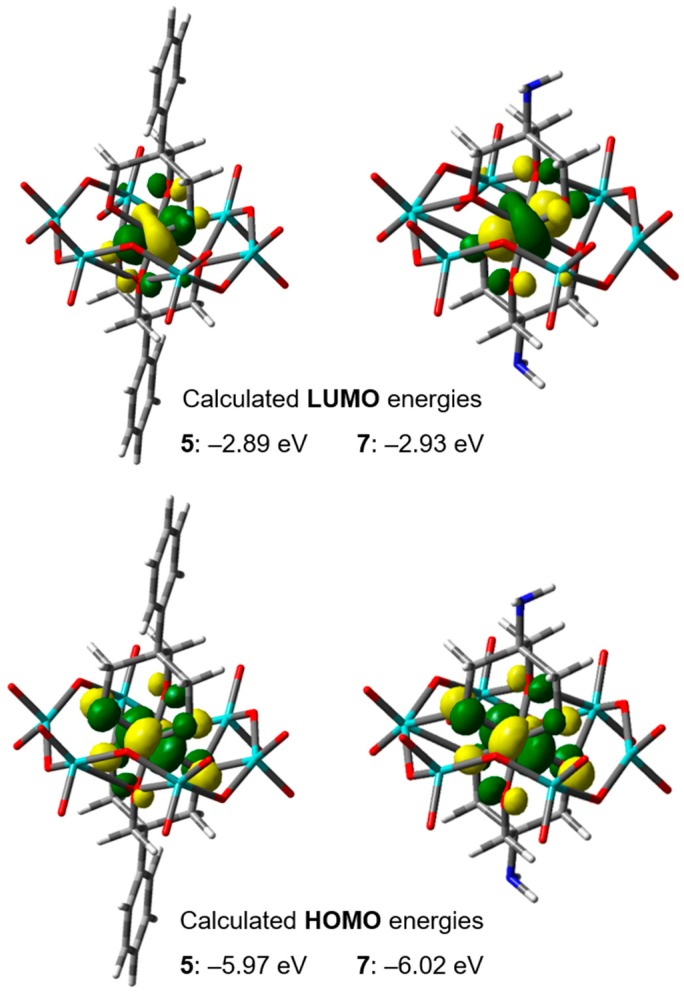
Representation of the frontier molecular orbitals for the geometry optimized Anderson-type POM derivatives **5** and **7**.

As pointed out above, the linkage of the photosensitizer moieties to the POMs via C–C bonds is expected to offer increased chemical stability in comparison to the related dyads, e.g., featuring hydrolytically more labile imine linkages. However, the electronic structure of the polyanions should not (or only marginally) be affected by the nature of the covalent bond to the PS. To verify this assumption, the frontier molecular orbitals (FMOs), i.e., the highest occupied and lowest unoccupied molecular orbitals (HOMO and LUMO, respectively), of the phenyl-equipped Anderson-type POM **5**, were calculated. The DFT calculation was carried out on the geometry-optimized structure of **5** using the B3LYP functional (details on the computational methodology are given in the Experimental Section). The derived values were compared to the HOMO and LUMO energies reported recently by Schönweiz et al. for some TRIS-functionalized Anderson-type POMs [10]. It was proposed that the first one-electron reduction of these clusters would occur at the central metal ion, i.e., reduction from the +III to the +II state. This process, arising from a photoinduced electron transfer from the PS to the POM, is believed to contribute to the overall HER activity found for these PS-POM dyads. The calculated frontier molecular geometries and the HOMO/LUMO energies of **5** in comparison to the Mn(III)-centered TRIS-functionalized Anderson-type POM (**7**, Scheme 2) are depicted in Figure 6. The computed closed-shell ground-state configurations of **5** and **7** feature basically the same localization of the FMOs, which is in full agreement with the results presented by Streb and co-workers (the deviation of the calculated HOMO/LUMO energies is likely due to differences in the applied computational methods) [10]. Based on these findings, it is concluded that the electronic structure of the Anderson-type cluster unit is hardly affected by the lateral substituent.

#### 2.4.2. Electrochemical Properties

**Figure 7:**
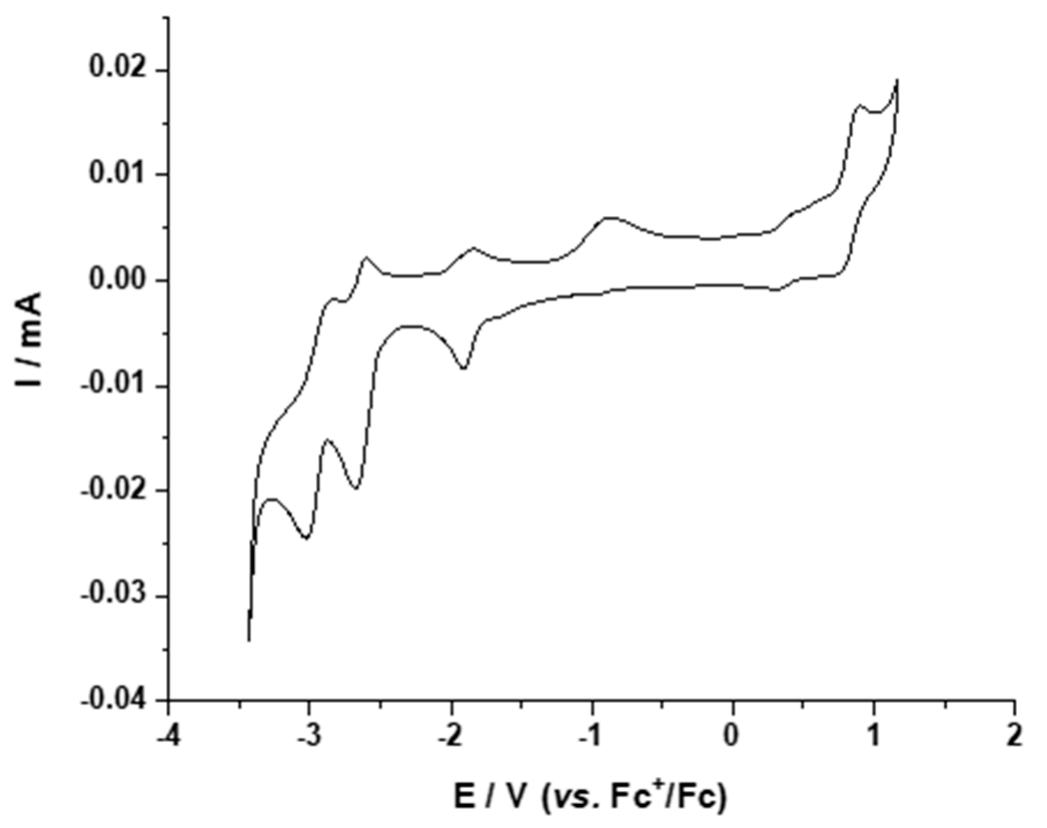
Cyclic voltammetry (CV) curve for **11**. The measurement was performed at a scan rate of 200 mV/s in DMF (containing 0.1 (TBA)PF_6_).

As for the compounds possessing the Wells–Dawson-type POM unit, the electrochemical properties of the Anderson-type POM derivatives were also studied by CV and SWV. However, for solubility reasons, DMF (containing 0.1 M TBAF as supporting electrolyte) had to be used as a solvent in all cases. The phenyl-decorated POM **5** exhibited two reversible redox processes at 0.24 V and −1.34 V which correspond to the Mn^IV^/Mn^III^ and Mn^III^/Mn^II^ redox couple, respectively (for the latter, there is some inconsistency in the literature—this redox process has also been assigned to the Mo^VI^/Mo^V^ couple, Figure SM-32) [10,18]. The bpy-functionalized counterpart **6** revealed basically the same electrochemical behavior, but with the redox potentials being shifted to 0.39 and −1.17 V, respectively (attributed to the different electronic nature of the aromatic substituent, Figure SM-33a/c). At more negative potentials, an additional reduction signal appeared for **6** (at −2.71 V). This signal decreased in intensity with the increasing number of CV cycles; thus, degradation of the cluster due to the reduction of the Mo(VI) centers is proposed under these conditions (Figure SM-33b). However, the redox process associated with the bpy^⋅ –^/bpy couple could not be identified. The redox potentials of the Rh(III)- and Ir(III)-containing dyads (**10** and **11**) are summarized in Table 2. In addition to the aforementioned redox processes arising from the POM unit, the typical oxidation and reduction processes associated with the attached metal complexes could also be observed. The Ir(III)-containing dyad **11**, for example, showed the typical Ir^IV^/Ir^III^ redox couple at 0.83 V; moreover, the ligand-centered reductions (ppy/ppy^−^ bpy/bpy^−^) and were identified at negative potentials (Figure 7).

#### 2.4.3. UV/vis Absorption and Emission Properties

**Figure 8:**
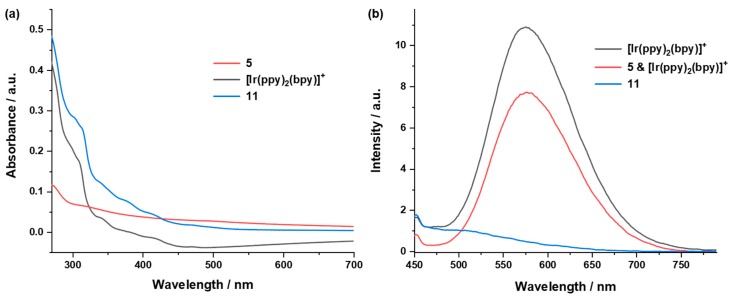
UV/vis absorption (**a**) and emission spectra (**b**) of **5**, [Ir(ppy)_2_(bpy)]^+^ and **11** (all spectra were measured at room temperature in DMF (c = 10^−5^ mol L^−1^).

UV/vis absorption and emission spectroscopy were employed to obtain a first insight into the photophysical properties of the dyads based on the bpy-functionalized Anderson-type POM **6**. As already pointed out in the context of the dyads **8** and **9** (see Section 2.4.2), the UV/vis absorption spectra of **10** and **11** reflected the absorption behavior of the subunits, i.e., the central POM and the attached Rh(III) or Ir(III) complexes. The only marginal difference in the position and shape of the bands pointed to a negligible electronic interaction in the ground state (Figure 8a). As for the Ir(III)-containing dyad **9**, no room-temperature emission was detected for **11**. This behavior was rationalized by an intramolecular electron-transfer from the photo-excited Ir(III) centers to the POM moieties [26]. As shown by the control experiment, the intermolecular emission quenching was by far less efficient: The mixture of **5** and [Ir(ppy)_2_(bpy)]^+^, in a 1:2 ratio, revealed an emission at 592 nm with a relative intensity of ca. 70% compared to that of the [Ir(ppy)_2_(bpy)]^+^ reference (Figure 8b). Currently, in-depth photophysical studies of compound **11**, including time-resolved measurements, are ongoing. With respect to a potential application of **11** as photocatalyst in the HER, a fundamental understanding of the photophysical (and electrochemical) properties is required. This comparative study, also taking the analogous Ir(III)-containing dyad reported by Streb et al. into account [10,18], will be published elsewhere. As pointed out above, quenching studies using the Rh(III)-containing dyad 10 were obsolete, due to the non-emissive character of [Rh(ppy)_2_(bpy)]^+^ at room temperature.

## 3. Materials and Methods

All reagents and analytical-grade solvents were purchased from commercial suppliers and used as received. In detail, all solvents were obtained from Merk KGaA (Darmstadt, Germany); for the reagents the suppliers were as follows: Phenylacetaldehyde, (TBA)Br, (TBA)OH, NaVO_3_, MoO_3_, Mn(OAc)_3_, *trans*-crotonaldehyde, formalin solution, 2,2′-bipyridine (all from Merk KGaA, Darmstadt, Germany), 2-phenylpyridine, IrCl_3_ × *3*H_2_O (all from TCI Deutschland GmbH, Eschborn, Germany), RhCl_3_ × *x*H_2_O (VEB Bergbau- und Hüttenkombinat “Albert Funk”, Halsbrücke, Germany). 2-(Hydroxymethyl)-2-phenyl-1,3-propanediol (**1**) [30], 4-methyl-2,2′-bipyridine [32], (TBA)_3_H_5_K[P_2_W_15_V_3_O_62_] [34], (TBA)_4_[Mo_8_O_26_] [35], [(ppy)_2_RhCl]_2_ [44], and [(ppy)_2_IrCl]_2_ [45] were prepared according to literature reports. Reactions under microwave irradiation were carried out in closed vials in a Initiator-8 MW (Biotage EU, Uppsala, Sweden). NMR spectra were recorded with a 400-MHz Avance instrument (Bruker BioSpin, Rheinstetten, Germany) in deuterated solvents (Euriso-Top GmbH, Saarbrücken, Germany) at 25 °C. Chemical shifts are given in ppm and referenced to the solvent signal. Elemental analyses were carried out utilizing an EA3000 elemental analyzer (EuroVector s.p.A., Milan, Italy). ESI and MALDI time-of-flight mass spectra were measured on a Bruker-(Q)-TOF microTOF II and a Bruker ultraflex III instrument, respectively (Bruker Daltonik, Bremen, Germany). For the latter, *trans*-2-[3-(4-*tert*-butylphenyl)-2-methyl-2-propenylidene]malononitrile (commonly abbreviated as DCTB; Merck KGaA, Darmstadt) and 9-aminoacridine (9AA; Merck KGaA, Darmstadt) were employed as matrix compounds. X-ray photoelectron spectroscopy (XPS) was performed in a UHV Multiprobe system equipped with a monochromatic X-ray source (Al-K_α_) and an electron analyzer (Argus CU) with an energy resolution of 0.6 eV (Scienta Omicron, Taunusstein, Germany). The samples were prepared by drop-casting a CH_3_CN solution of the material (2 µL) onto Au substrates, which were precleaned by O_2_-plasma treatment. Afterward, the samples were dried under ambient conditions and introduced into the UHV system for measurements. For quantitative analysis, the secondary electron background was subtracted from the high-resolution spectra, and the peaks were fitted using Voigt functions. UV/vis absorption spectra were recorded on a Lambda-750 spectrometer in 10^−5^ M solutions (PerkinElmer, Rodgau, Germany). Emission spectra at room temperature were acquired with an FLS980 spectrometer (Edinburgh Instruments Ltd., Lvingston, U.K.) using solutions with optical densities of 0.3 at 500 nm excitation wavelength. Cyclic voltammetry (CV) measurements were carried out using a Autolab PGSTAT30 potentiostat (Metrohm, Filderstadt, Germany), with a standard three-electrode configuration, by applying a glassy-carbon working electrode, a platinum-rod auxiliary electrode and an AgCl/Ag reference electrode. The experiments were carried out in degassed solvents (HPLC grade) containing 0.1 M (TBA)PF_6_ salt. At the end of each measurement, ferrocene was added as an internal standard. The same experimental setup was also used for the square-wave voltammetry (SWV). All these measurements were executed with ferrocene, as an internal standard, using the following parameters: P_H_ = 5, P_W_ = 150, and S_H_ = −10.

*2-([2,2′-Bipyridin]-4-yl)-2-(hydroxymethyl)propane-1,3-diol (bpy-triol, 2).* 4-Methyl-2,2′- bipyridine (1 g, 5.87 mmol) and KOH (20 mg, 0.36 mmol) were filled into a microwave vial. Formalin solution (37 wt-%, 10 mL) was added, and the mixture was stirred for 10 h at 130 °C under microwave irradiation. In the course of the reaction, the pressure inside the vial was raised to 14 bar. After cooling, the reaction mixture was evaporated to dryness. Methanol (30 mL) was added and evaporated to remove the remaining formaldehyde (this step of the work-up procedure was repeated). The solid was redissolved in methanol (30 mL); concentrated HCl was added until the solution showed a pH value of 1 (ca. 20 drops), and the solution was stirred overnight under reflux. Afterward, the solvent was evaporated to yield a red solid, which was suspended in ethanol (5 mL). The white hydrochloride precipitate was filtered off and was subsequently washed with ethanol and diethyl ether (each 5 mL). The filter residue was redissolved in water (10 mL), and solid Na_2_CO_3_ was added until the solution became basic (pH value of ca. 10). The white precipitate was isolated and washed with ethanol and diethyl ether (each 5 mL) to yield **2** as a white solid. ^1^H NMR (400 MHz, CD_3_OD, *δ*): 8.67 (ddd, *J* = 4.9, 1.8, 1.0 Hz, 1H), 8.59 (dd, *J* = 5.3, 0.8 Hz, 1H), 8.39 (dd, *J* = 2.0, 0.8 Hz, 1H), 8.27 (dt, *J* = 8.1, 1.1 Hz, 1H), 7.96 (td, *J* = 7.8, 1.8 Hz, 1H), 7.58 (dd, *J* = 5.4 Hz, 1.9 Hz, 1H), 7.46 (ddd, *J* = 7.6, 4.9, 1.2 Hz, 1H), 4.00 (s, 6H) ppm. ^13^C NMR (100 MHz, CD_3_OD, *δ*): 156.1, 155.3, 153.2, 148.8, 148.5, 137.5, 123.9, 123.7, 121.5, 120.9, 63.2, 50.0 ppm. Elem. anal. calcd. for C_14_H_16_O_3_N_2_Na: C 59.39, H 5.69, N 9.89; found C 59.83, H 5.69, N 10.01. HR-ESI MS ESI-MS (*m/z*) calcd. for C_14_H_16_N_2_ 261.117; found 261.131.

*(TBA)_5_H_4_[P_2_W_15_V_3_O_62_{C_10_H_11_}] **(3).*** A nitrogen-purged solution of **1** (42.3 mg, 0.23 mmol) and (TBA)_3_H_5_K[P_2_W_15_V_3_O_62_] (900 mg, 0.184 mmol) in *N*,*N*-dimethylacetamide (5 mL) was heated at 80 °C for eight days. The addition of an excess of diethyl ether to the solution resulted in the precipitation of an orange solid. The crude product was dissolved in acetonitrile (2 mL) and precipitated again in excess diethyl ether (this process was repeated three times). The product was then collected, washed with diethyl ether (3 × 10 mL), and dried *in vacuo*. Yield: 399 mg (41% based on the precursor cluster). ^1^H NMR (400 MHz, CD_3_CN, *δ*): 7.52–7.39 (m, 4H), 7.32 (s, 1H), 5.78 (s, 6H), 3.20–3.10 (m, 40H), 1.71–1.56 (m, 40H), 1.44 (h, *J* = 7.3 Hz, 40H), 0.98 (t, *J* = 7.3 Hz, 60H) ppm. ^31^P NMR (162 MHz, CD_3_CN, *δ*): −7.42, −13.51 ppm. ^13^C NMR (101 MHz, CD_3_CN, *δ*): 129.9, 127.1, 90.5, 59.33, 24.4, 20.4, 13.9 ppm. HR-ESI MS (*m/z*): calcd. for {(TBA)_3_H[P_2_W_15_V_3_O_62_C_10_H_11_]}^3−^ 1608.1773; found 1608, 2256.

*(TBA)_5_H[P_2_W_15_V_3_O_62_{C_14_H_13_N_2_}]***(4).** A nitrogen-purged solution of **2** (35.5 mg, 0.136 mmol) and (TBA)_5_H_4_[P_2_W_15_V_3_O_62_] (707 mg, 0.136 mmol) in *N*,*N*-dimethylacetamide (4 mL) was heated at 80 °C for eight days. The product was precipitated by addition to an excess of diethyl ether, collected, dissolved in CH_3_CN (5 mL) and reprecipitated in excess diethyl ether (the dissolving–precipitation process was repeated three times). The product was then collected, washed with diethyl ether (3 × 10 mL), and dried *in vacuo*. Yield: 305 mg (42% based on the precursor cluster). ^1^H NMR (400 MHz, CD_3_CN, *δ*): 8.70 (t, *J* = 4.8 Hz, 2H), 8.54 (d, *J* = 1.8 Hz, 1H), 8.45 (dt, *J* = 7.9, 1.1 Hz, 1H), 7.92 (td, *J* = 7.7, 1.8 Hz, 1H), 7.47 (dd, *J* = 5.2 Hz, 1.9 Hz, 1H), 7.42 (ddd, *J* = 7.5, 4.8, 1.2 Hz, 1H), 5.86 (s, 6H), 3.22–3.13 (m, 40H), 1.72–1.65 (m, 40H), 1.46–1.38 (m, 40H), 1.01 (t, *J* = 7.3 Hz, 60H) ppm. ^31^P NMR (162 MHz, CD_3_CN, *δ*): −7.40, −13.48 ppm. MALDI MS (*m/z*): calcd. for {(TBA)_4_H[P_2_W_15_V_3_O_62_C_14_H_13_N_2_] + 2 DCTB}^−^ 2943.13; found 2943.28

*(TBA)_3_[MnMo_6_O_24_{C_10_H_11_}_2_]***(5).** A mixture of (TBA)_4_Mo_8_O_26_ (500 mg, 0.232 mmol), Mn(III) acetate dihydrate (93 mg, 0.348 mmol) and **1** (148 mg, 0.813 mmol) in CH_3_CN (12 mL) was heated under reflux for 19 h. The orange solution was cooled to room temperature and filtered to remove a fine white solid. The diffusion of diethyl ether into the filtrate yielded large orange crystals. Yield: 455 mg (98% based on the precursor cluster). ^1^H NMR (400 MHz, CD_3_CN, *δ*): 63.14 (br s, 6H), 7.23 (br s, 4H), 7.03 (br s, 2H), 6.92 (br s, 4H), 3.22–3.05 (m, 24H), 1.63–1,52 (m, 24H), 1.37–1.29 (m, 24H), 0.93 (t, *J* = 4.0 Hz, 36H) ppm. HR-ESI MS: calcd. for {(TBA)H[MnMo_6_O_24_C_20_H_22_]}^−^ 1520.7161; found 1520.9817.

*(TBA)_3_[MnMo_6_O_24_{C_14_H_13_N_2_}_2_]***(6).** A mixture of (TBA)_4_Mo_8_O_26_ (165 mg, 0.076 mmol), Mn(III) acetate dihydrate (30 mg, 0.113 mmol) and **2** (70 mg, 0.270 mmol) in *N*,*N*-dimethylacetamide (3.5 mL) was stirred at 80 °C for 24 h to yield a deeply orange-colored solution. The orange solution was cooled to room temperature, and the product was obtained by vapor diffusion of diethyl ether into this solution. The orange crystals were isolated by filtration and thoroughly washed with diethyl ether. Yield: 183 mg (63% based on the precursor cluster). ^1^H NMR (400 MHz, d_6_-DMSO, *δ*): 64.21 (br s, 6H), 8.77 (d, *J* = 4.5 Hz, 2H), 8.45 (d, *J* = 7.8 Hz, 2H), 8.27 (s, 2H), 8.15 (br s, 2H), 7.96 (t, *J* = 7.7 Hz, 2H), 7.47 (dd, *J* = 7.5, 4.2 Hz, 2H), 7.10 (br s, 1H), 3.21–3.08 (m, 24H), 1.59–1.51 (m, 24H), 1.34–1.25 (m, 24H), 0.92 (t, *J* = 7.2 Hz, 36H) ppm. HR-ESI MS (*m/z*): calcd. for {MnMo_6_O_24_C_28_H_26_N_4_}^3–^ 478.1552; found 478.1563.

*General procedure for the complexation of the Wells–Dawson-type POM-bpy***conjugate 4.** A mixture of **4** and the respective μ-dichloro-bridged [(ppy)_2_MCl]_2_ precursor complex (M = Rh or Ir) in a CH_2_Cl_2_/methanol mixture (5:2 ratio) was heated under a nitrogen atmosphere to 55 °C for 12 h. The product was precipitated by the addition of diethyl ether (10 mL), filtered off, washed with diethyl ether and dried *in vacuo*.

*(TBA)_5_[P_2_W_15_V_3_O_59_(OCH_2_)_3_C(C_10_H_7_N_2_)Rh(C_11_H_8_N)_2_]***(8).** Following the general procedure for the complexation of Well–Dawson-type POM-bpy conjugate, **4** (46.5 mg, 8.4 µmol) and [(ppy)_2_RhCl]_2_ (3.8 mg, 4.2 µmol) were used. Yield: 22 mg (44%). ^1^H NMR (400 MHz, CD_3_CN, *δ*): 8.63 (d, *J* = 8.5 Hz, 1H), 8.43 (s, 1H), 8.13 (s, 1H), 8.05 (t, *J* = 7.6 Hz, 2H), 7.98 (d, *J* = 5.1 Hz, 1H), 7.95 (d, *J* = 5.7 Hz, 1H), 7.91 (d, *J* = 7.0 Hz, 1H), 7.83 (d, *J* = 7.9 Hz, 2H), 7.62 (s, 1H), 7.59 (s, 1H), 7.47 (s, 1H), 7.09 (t, *J* = 7.5 Hz, 4H), 6.96 (t, *J* = 7.4 Hz, 2H), 6.28 (d, *J* = 7.7 Hz, 2H), 5.75 (s, 6H), 3.15–3.07 (m, 40H), 1.66–1.58 (m, 40H), 1.42–1.36 (m, 40H), 0.98 (t, *J* = 7.3 Hz, 60H) ppm. MALDI-MS (*m/z*) calcd. for {(TBA)_4_[P_2_W_15_V_3_O_62_C36H29N4Rh} 5555.01; found 5555.69.

*(TBA)_5_[P_2_W_15_V_3_O_59_(OCH_2_)_3_C(C_10_H_7_N_2_)Ir(C_11_H_8_N)_2_]***(9).** Following the general procedure for the complexation of the Well–Dawson-type POM-bpy conjugate, **4** (46.5 mg, 8.4 µmol) and [(ppy)_2_IrCl]_2_ (4.5 mg, 4.2 µmol) were used. Yield: 17 mg (36%). ^1^H NMR (400 MHz, CD_3_CN, *δ*): 8.63 (d, *J* = 8.5 Hz, 1H), 8.43 (s, 1H), 8.13 (s, 1H), 8.05 (t, *J* = 7.6 Hz, 2H), 7.98 (d, *J* = 5.1 Hz, 1H), 7.95 (d, *J* = 5.7 Hz, 1H), 7.91 (d, *J* = 7.0 Hz, 1H), 7.83 (d, *J* = 7.9 Hz, 2H), 7.62 (s, 1H), 7.59 (s, 1H), 7.47 (s, 1H), 7.09 (t, *J* = 7.5 Hz, 4H), 6.96 (t, *J* = 7.4 Hz, 2H), 6.28 (d, *J* = 7.7 Hz, 2H), 5.72 (s, 6H), 3.15–3.07 (m, 40H), 1.66–1.58 (m, 40H), 1.43–1.36 (m, 40H), 0.98 (t, *J* = 7.3 Hz, 60H) ppm. HR-ESI MS (*m/z*) calcd. for {P_2_W_15_V_3_O_60_C_36_H_29_ N_4_Ir} 1547.85; found 1547.98.

*General procedure for the complexation of the Anderson-type POM-bpy***conjugate 5.** A mixture of **5** and the respective μ-dichloro-bridged [(ppy)_2_MCl]_2_ precursor complex (M = Rh or Ir) in acetonitrile (10 mL) was heated in the dark and under a nitrogen atmosphere to 82 °C for 3 h. After cooling to room temperature, the solvent volume was reduced to 2 mL under reduced pressure. Slow diffusion of diethyl ether into this solution afforded the products which were filtered off, washed with diethyl ether (10 mL) and dried *in vacuo*.

*(TBA)[MnMo_6_O_18_{(OCH_2_)_3_C(C_10_H_7_N_2_)Rh(C_11_H_8_N)_2_}_2_]***(10).** Following the general procedure for the complexation of the Anderson-type POM-bpy conjugate, 5 (42.9 mg, 20.8 µmol) and [(ppy)_2_RhCl]_2_ (18.6 mg, 20.8 µmol) were used. Yield: 22 mg (43%). ^1^H NMR (400 MHz, DMSO-*d*_6_, *δ*): 63.7 (br s, 6H), 8.84 (s, 2H), 8.24 (d, *J* = 7.9 Hz, 8H), 7.95 (br s, 10H), 7.86 (s, 2H), 7.71 (s, 2H), 7.64 (t, *J* = 5.6 Hz, 2H), 7.54 (s, 2H), 7.47 (s, 2H), 7.18 (br s, 4H), 7.07 (s, 4H), 6.94 (q, *J* = 6.8 Hz, 4H), 6.20 (dd, *J* = 9.5, 7.4 Hz, 4H), 3.14 (d, *J* = 9.9 Hz, 8H), 1.55 (s, 8H), 1.30 (q, *J* = 7.5 Hz, 8H), 0.92 (t, *J* = 7.2 Hz, 12H) ppm. MALDI-MS (*m/z*) calcd. for {[MnMo_6_O_22_C_50_H_36_N_8_Rh] + 9AA}^−^ 1216.95; found 1217.31.

*TBA[MnMo_6_O_18_{(OCH_2_)_3_C(C_10_H_7_N_2_)Ir(C_11_H_8_N)_2_}_2_]***(11).** Following the general procedure for the complexation of the Anderson-type POM-bpy conjugate, 5 (40 mg, 19.4 µmol) and [(ppy)_2_IrCl]_2_ (20.8 mg, 19.4 µmol) were used. Yield: 17 mg (33%). ^1^H NMR (400 MHz, DMSO-*d*_6_, *δ*): 63.9 (br s, 6H), 8.82 (s, 2H), 8.26 (d, *J* = 8.2 Hz, 8H), 7.98 (br s, 10H), 7.88 (s, 2H), 7.74 (s, 2H), 7.64 (t, *J* = 6.4 Hz, 2H), 7.56 (s, 2H), 7.50 (s, 2H), 7.26–7.13 (m, 4H), 7.07 (q, *J* = 6.9 Hz, 4H), 6.96 (q, *J* = 8.1 Hz, 4H), 6.22 (dd, *J* = 9.1, 7.6 Hz, 4H), 3.22–3.12 (m, 8H), 1.60–1.55 (m, 8H), 1.35–1.29 (m, 8H), 0.94 (t, *J* = 7.3 Hz, 12H) ppm. MALDI-MS (*m/z*) calcd. for {(TBA)_2_[MnMo_6_O_26_C_50_H_36_N_8_Ir]}^+^ 2919.36; found 2918.92.

*Crystal Structure Determination.* The intensity data of **5** and **6** were collected on a Nonius KappaCCD diffractometer using graphite-monochromated Mo-K_α_ radiation (Bruker AXS, Karlsruhe, Germany). Data were corrected for Lorentz and polarization effects; absorption was considered on a semi-empirical basis using multiple-scans [46,47,48]. The structures were solved by direct methods (SHELXS) and refined by full-matrix least-squares techniques against Fo^2^ (SHELXL-97) [49]. All hydrogen atoms were included at calculated positions with fixed thermal parameters. All non-disordered, non-hydrogen atoms were refined anisotropically [49]. Mercury was used for structure representations [50].

*Crystal Data for***5**: C_72_H_136_MnMo_6_N_5_O_24_, Mr = 2086.44 g mol^−1^, pale pink prism, size 0.134 × 0.112 × 0.102 mm^3^, monoclinic, space group C2/c, a = 30.0091(5), b = 17.0856(3), c = 21.9984(3) Å, β = 128.733(1)°, V = 8798.5(2) Å3, T= −140 °C, Z = 4, ρ_calcd._ = 1.575 g cm^−3^, µ(Mo-K_α_) = 10.37 cm^−1^, multi-scan, transmin: 0.6749, transmax: 0.7456, F(000) = 4288, 50,113 reflections in h(−38/38), k(−21/22), l(−28/25), measured in the range 2.29° ≤ Θ ≤ 27.48°, completeness Θ_max_ = 99.8%, 10,060 independent reflections, R_int_ = 0.0287, 9148 reflections with Fo > 4σ(Fo), 582 parameters, 0 restraints, R1_obs_ = 0.0243, wR^2^_obs_ = 0.0536, R1_all_ = 0.0286, wR^2^_all_ = 0.0556, GOOF = 1.068, largest difference peak and hole: 0.578/−0.425 e Å^−3^.

*Crystal Data for***6**: C_88_H_161_MnMo_6_N_10_O_27_, Mr = 2421.85 gmol^−1^, orange prism, size 0.108 × 0.098 × 0.084 mm^3^, orthorhombic, space group Pca2_1_, a = 17.4313(2), b = 22.2868(2), c = 27.0044(3) Å, V = 10490.88(19) Å^3^, T= −140 °C, Z = 4, ρ_calcd._ = 1.533 gcm^−3^, µ (Mo-K_α_) = 8.85 cm^−1^, multi-scan, transmin: 0.7120, transmax: 0.7456, F(000) = 5008, 104,170 reflections in h(−22/22), k(−28/26), l(−35/32), measured in the range 1.76° ≤ Θ ≤ 27.48°, completeness Θ _max_ = 99.6%, 23,456 independent reflections, R_int_ = 0.0347, 21,642 reflections with Fo > 4σ(Fo), 1205 parameters, 3 restraints, R1_obs_ = 0.0341, wR^2^_obs_ = 0.0716, R1_all_ = 0.0394, wR^2^_all_ = 0.0747, GOOF = 1.037, Flack-parameter 0.398(18), largest difference peak and hole: 0.972/−0.584 e Å^−3^.

*Computational methodology.* The theoretical calculations based on density functional theory (DFT) were performed with the Gaussian-16 program package (version A.03) [51]. The standard B3LYP functional was selected [52,53]. The 6-31G** basis set was used for all atoms (C, H, O, N) except for Mo and Mn, which were described by an effective core potential and the associated orbitals (LANL2DZ). For all calculations, the solvent environment was modeled for DMF using the implemented polarization continuum model (PCM) [54,55]. The corresponding geometries of the singlet ground states were optimized from reasonable initial estimates. In cases of difficult SCF convergence, additional extra quadratic (xqc) functions were used. The true nature of all minima structures was confirmed by vibrational analysis showing no imaginary frequencies. The graphical visualizations of the 3D representations were generated by GaussView (version 6.0.16).

## 4. Conclusions

The tris-alkoxylation of two appropriate POM precursors with a triol-functionalized bpy derivative has yielded the corresponding organo-functionalized POM hybrids. The pending bpy units have subsequently been used for the binding of transition metal complexes, namely a [Rh(ppy)_2_]^+^ and an [Ir(ppy)_2_]^+^ fragment. These dyads have been characterized by NMR and XPS spectroscopy, as well as mass spectrometry. Basic photophysical and electrochemical studies have been conducted and indicated a negligible electronic interaction of the subunits, i.e., the POM and the metal complex, in the ground state. Moreover, emission quenching studies on the Ir(III)-containing systems have suggested an efficient intramolecular electron transfer from the photo-excited Ir(III) centers to the POM units. In-depth photophysical studies of these assemblies, in comparison to Streb’s Ir(III) PS-POM conjugate [10,18], are currently ongoing. Fundamental knowledge of the photophysical processes in such dyads is of significant importance to understand their photocatalytic activity in the HER. In this respect, the chemically more robust dyad **11** is expected to be superior to the related imine-based dyad [10,18], in particular under hydrolytic conditions (e.g., using DMF/water mixtures, as solvents, and/or AcOH, as proton source). A comparative study taking these aspects into account is also the subject of current research.

## Figures and Tables

**Table 1:** Relative elemental composition as determined by XPS measurements.

Compound	Rh-to-P Ratio ^1^	Ir-to-P Ratio ^1^	Rh-to-Mo Ratio ^1^	Ir-to-Mo Ratio ^1^
**8**	1:(2.3 ± 0.2)	–	–	–
**9**	–	1:(2.1 ± 0.2)	–	–
**10**	–	–	1:(2.9 ± 0.2)	–
**11**	–	–	–	1:(3.1 + 0.2)

^1^ The calculated M:P and M:Mo ratios are 1:2 and 1:3, respectively.

**Table 2:** Redox potentials of the phenyl-equipped hybrids (**3** and **5**) and the Rh(III)- or Ir(III)-containing dyads (**8** to **11**) ^1^.

Compound	Oxidation Potentials (V vs. Fc^+^/Fc)	Reduction Potentials (V vs. Fc^+^/Fc)
**3**	–	−0.32, −0.62, −1.37, −1.83 ^2^
**8**	–	−1.35, −1.95, −2.68 ^2^
**9**	–	−1.23, −1.60, −1.91, −2.02, −2.81 ^2^
**5**	0.24 ^3^	−1.34 ^3^
**6**	0.39 ^3^	−1.28, −2.71 ^3^
**10**	0.42 ^3^	−1.09, −1.94, −2.56, −2.88 ^3^
**11**	0.86, 0.42 ^3^	−1.23, −1.85, −2.55, −2.91 ^3^

^1^ All redox potentials were determined at room temperature in degassed solutions; 0.1 M TBAPF_6_ was used as supporting electrolyte. Ferrocene was employed as an internal reference (Fc^+^/Fc redox couple at 0.45 V vs. SCE). All potentials were read out from the SWV measurements. ^2^ Acetonitrile as solvent. ^3^ DMF as solvent.

**Table 3:** UV/vis absorption data of the compounds based on Wells–Dawson-type POMS ^1^.

Compound	Absorption (wavelength/nm)
**[Rh(ppy)_2_(bpy)]^+^**	258, 297, 367^2^
**[Ir(ppy)_2_(bpy)]^+^**	307, 341, 380, 419^2^
**8**	258, 303, 370
**9**	258, 310, 380
**10**	260, 302, 369
**11**	310, 338, 378, 417

^1^ The dyads based on the Well–Dawson- and Anderson-type POMs were studied in CH_3_CN and DMF solution, respectively (for all measurements: c = 10^−5^ mol L^−1^). ^2^ The UV/vis absorption data correspond to DMF solutions (c = 10^−5^ mol L^−1^).

## Supplementary Materials

NMR and mass spectra of all synthesized compounds, the XPS data of all POM-containing dyads, the output files of the computational studies as well as additional electrochemical and photophysical spectra are available online.
