# Supplementary material for: Towards Covalent Photosensitizer-Polyoxometalate Dyads-Bipyridyl-Functionalized Polyoxometalates and Their Transition Metal Complexes
Source: Molecules. 2019 Dec 4;24(24):4446. doi: 10.3390/molecules24244446 (PMC6943497; doi:10.3390/molecules24244446)
Supplement: Supplementary file 1 [file molecules-24-04446-s001.pdf]

Supporting Information

Towards Covalent Photosensitizer-Polyoxometalate Dyads-Bipyridyl-Functionalized Polyoxometalates and Their Transition Metal Complexes

Andreas Winter [1,2], Patrick Endres [1,2], Erik Schröter [1,2], Michael Jäger [1,2], Helmar Görls [3], Christof Neumann [2,4], Andrey Turchanin [2,4] and Ulrich S. Schubert [*,1,2]

1 Laboratory of Organic and Macromolecular Chemistry (IOMC), Friedrich Schiller University Jena, Humboldtstr. 10, 07743 Jena, Germany; andreas.winter@uni-jena.de (A.W.); patrick.endres@uni-jena.de (P.E.); erik.schroeter@uni-jena.de (E.S.); michael.jager.iomc@uni-jena.de (M.J.)

2 Center for Energy and Environmental Chemistry (CEEC) Jena, Friedrich Schiller University Jena, Philosophenweg 7a, 07743 Jena, Germany; christof.neumann@uni-jena.de (C.N.); andrey.turchanin@uni-jena.de (A.T.)

3 Institute for Inorganic and Analytical Chemistry (IAAC), Friedrich Schiller University Jena, Humboldtstr. 8, 07743 Jena, Germany; helmar.goerls@uni-jena.de

4 Institute of Physical Chemistry (ICP), Friedrich Schiller University Jena, Lessingstr. 10, 07743 Jena, Germany

* Correspondence: ulrich.schubert@uni-jena.de; Tel.: +49-3641-948201

Table of content:

- NMR spectroscopy page 2
- Mass spectrometry page 10
- X-Ray photoelectron spectroscopy page 15
- Cyclic and square-wave voltammetry page 19
- UV/vis absorption and emission spectroscopy page 22

1. NMR spectroscopy

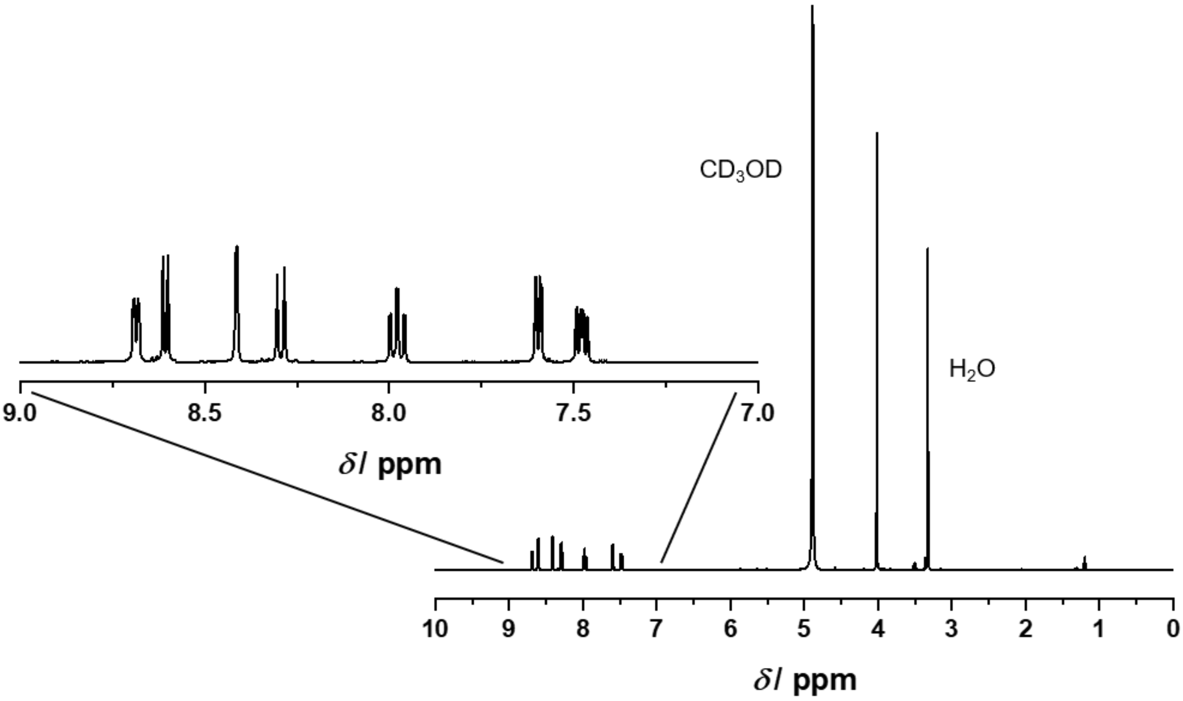

Figure SI- 1. ^{1}H NMR spectrum of **2** (400 MHz, CD_3OD).

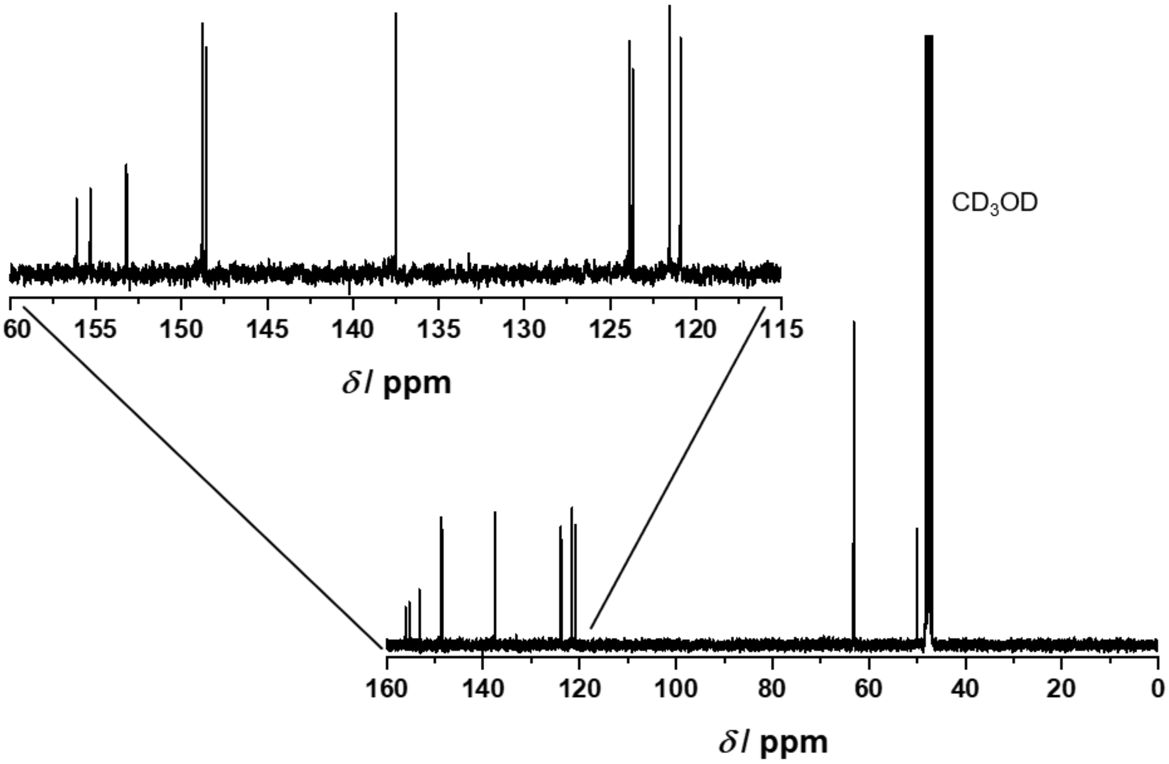

Figure SI- 2. ^{13}C NMR spectrum of **2** (101 MHz, CD_3OD).

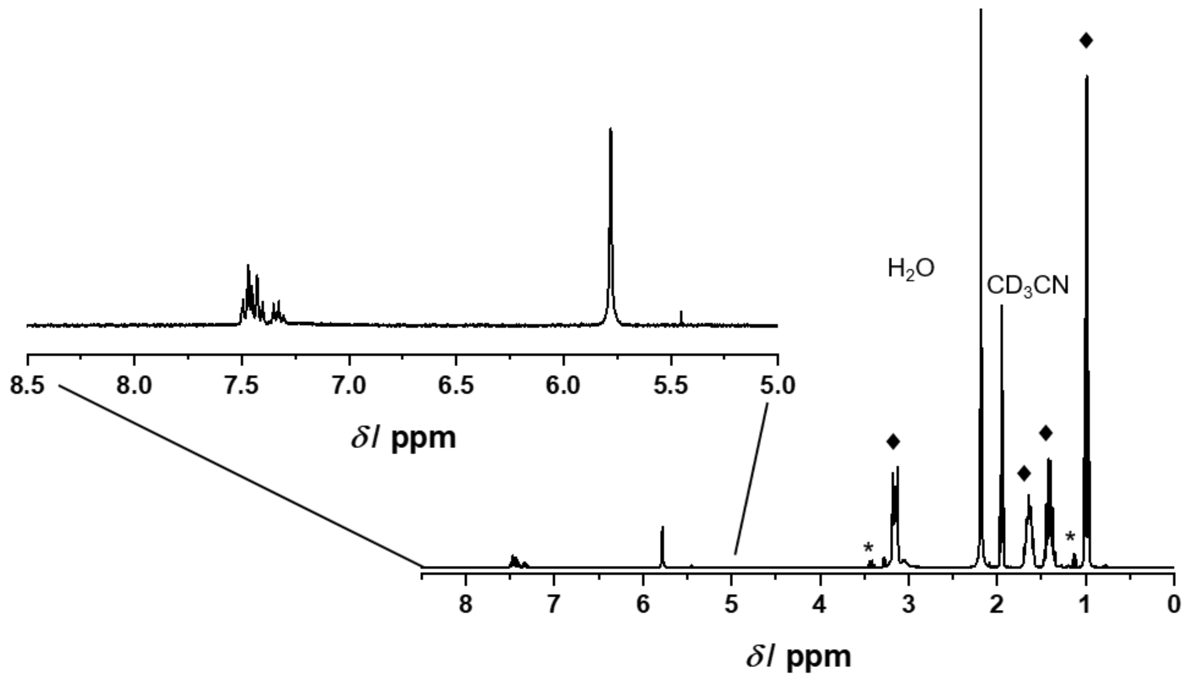

Figure SI- 3. ^{1}H NMR spectrum of **3** (400 MHz, CD_3CN). Residual diethyl ether (*) and TBA$^+$ signals (♦) are marked.

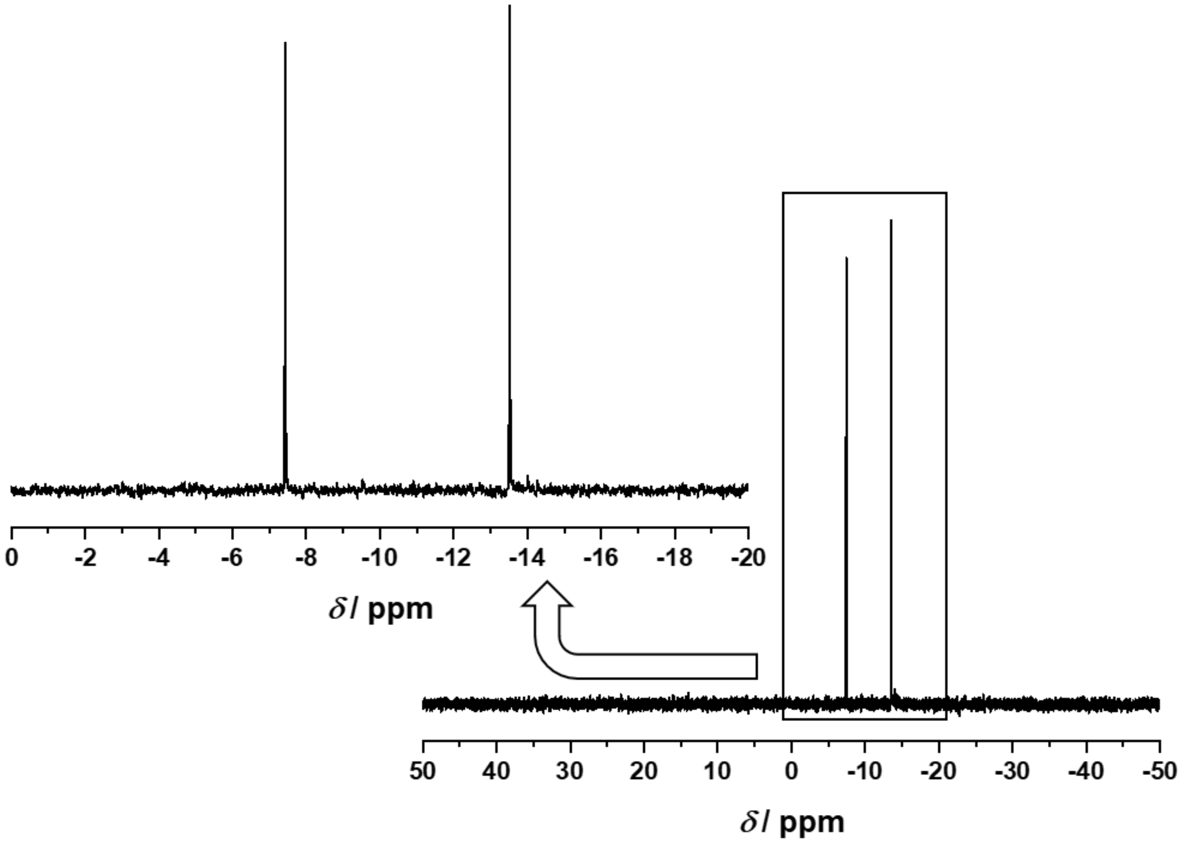

Figure SI- 4. ^{31}P NMR spectrum of **3** (162 MHz, CD_3CN).

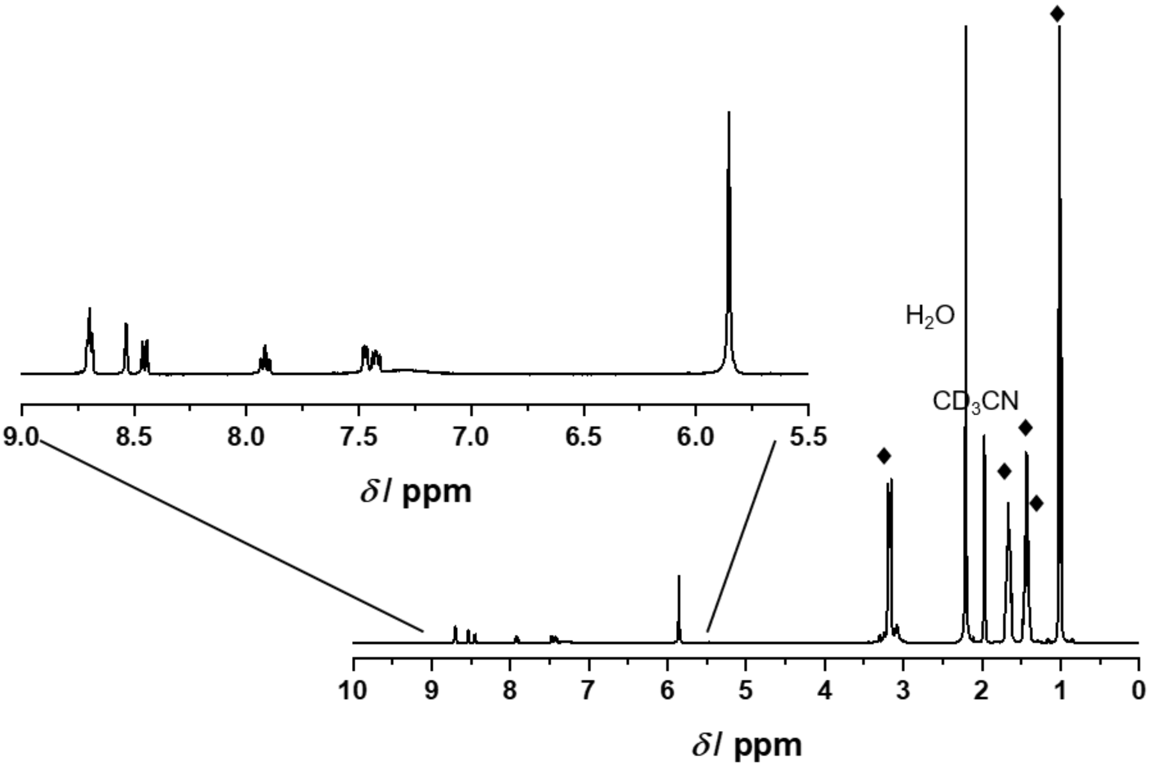

Figure SI- 5. ^{1}H NMR spectrum of **4** (400 MHz, CD_3CN). The TBA^+ signals (♦) are marked.

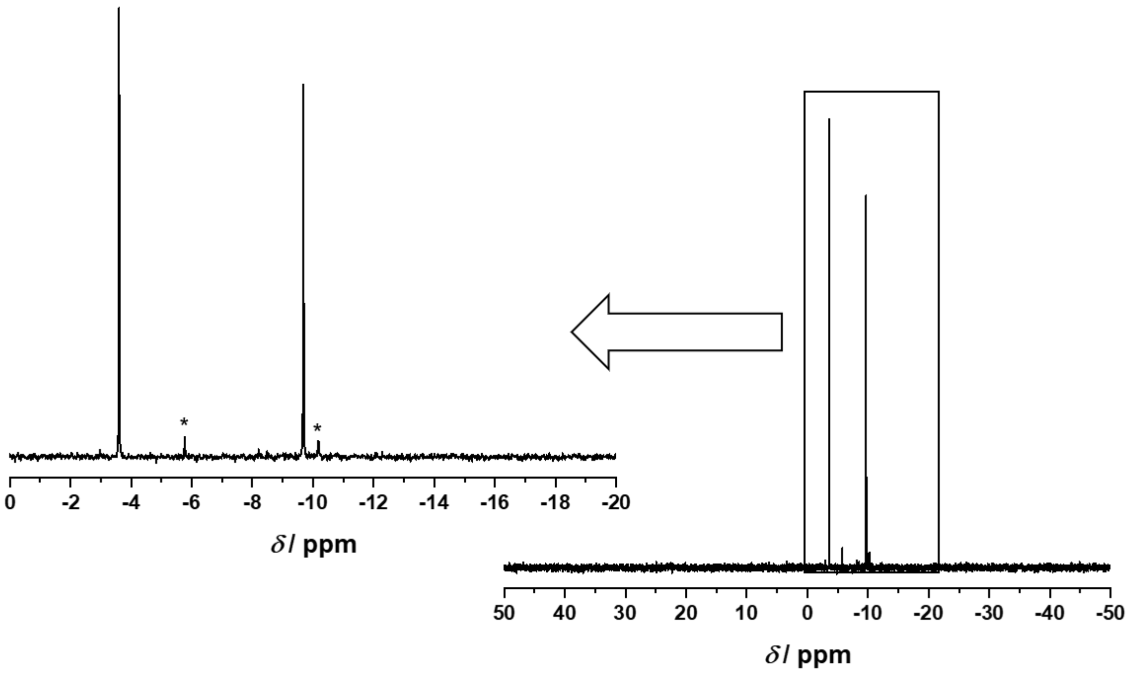

Figure SI- 6. ^{31}P NMR spectrum of **4** (162 MHz, CD_3CN). The signals arising from an unidentified cluster impurity are marked (*). Note, that this impurity could be removed after the subsequent complexation step (see Figures SI-8 and SI-9).

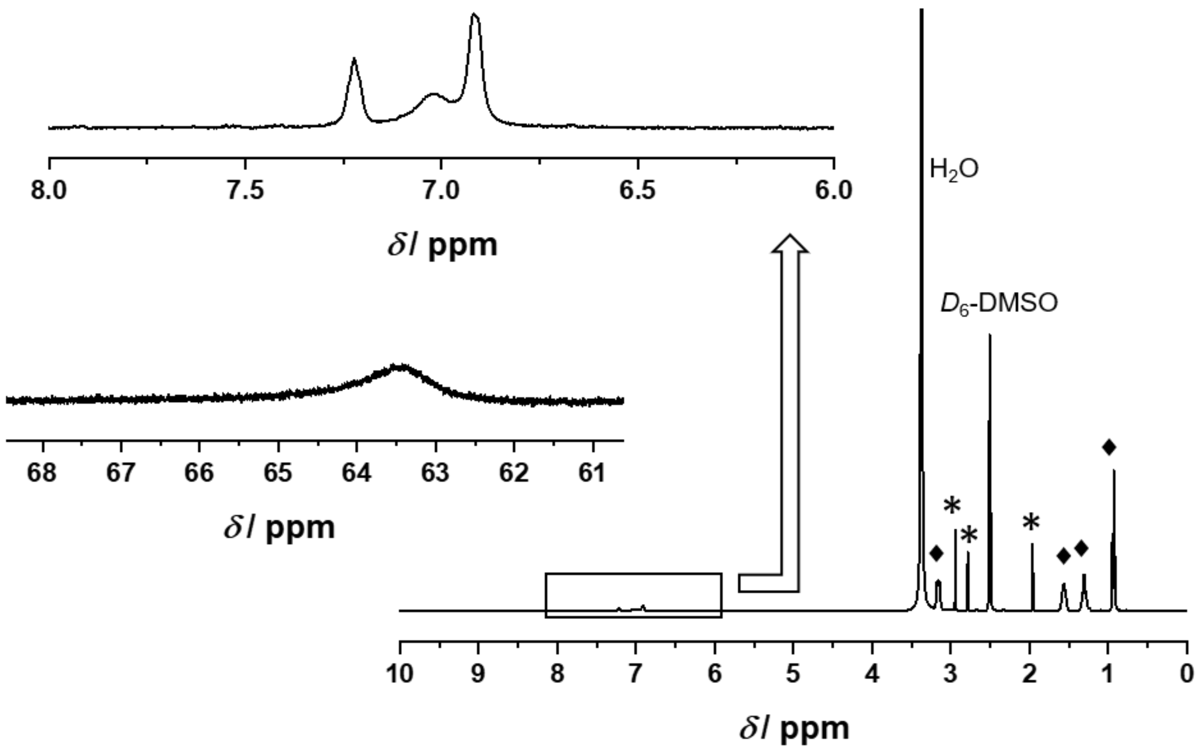

Figure SI- 7. ^{1}H NMR spectrum of **5** (400 MHz, *D*$_6$-DSMO). Residual DMAc (*) and TBA$^+$ signals (•) are marked.

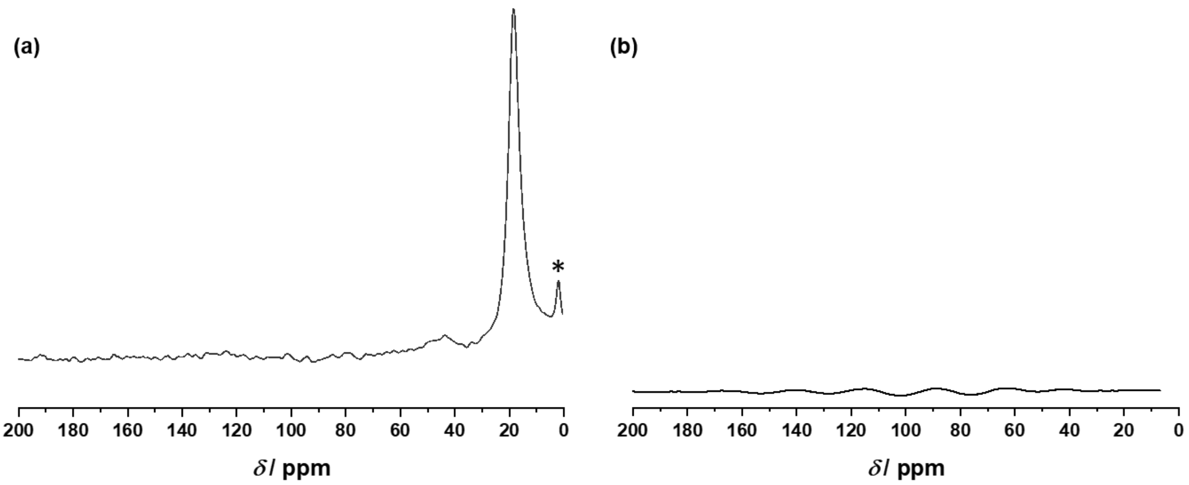

Figure SI- 8. ^{95}Mo NMR spectra of (TBA)$_4$[α-Mo$_8$O$_{26}$] (a) and **5** (b). The spectra were recorded at 26 MHz in D$_2$O and *D*$_6$-DMSO, respectively. In the case, of [α-Mo$_8$O$_{26}$]$^{4+}$, an artefact was observed at *ca.* 0 ppm (marked with an asterisk). Only the relevant ppm regime where resonances from {MO} clusters should appear were probed (0 to 200 ppm).

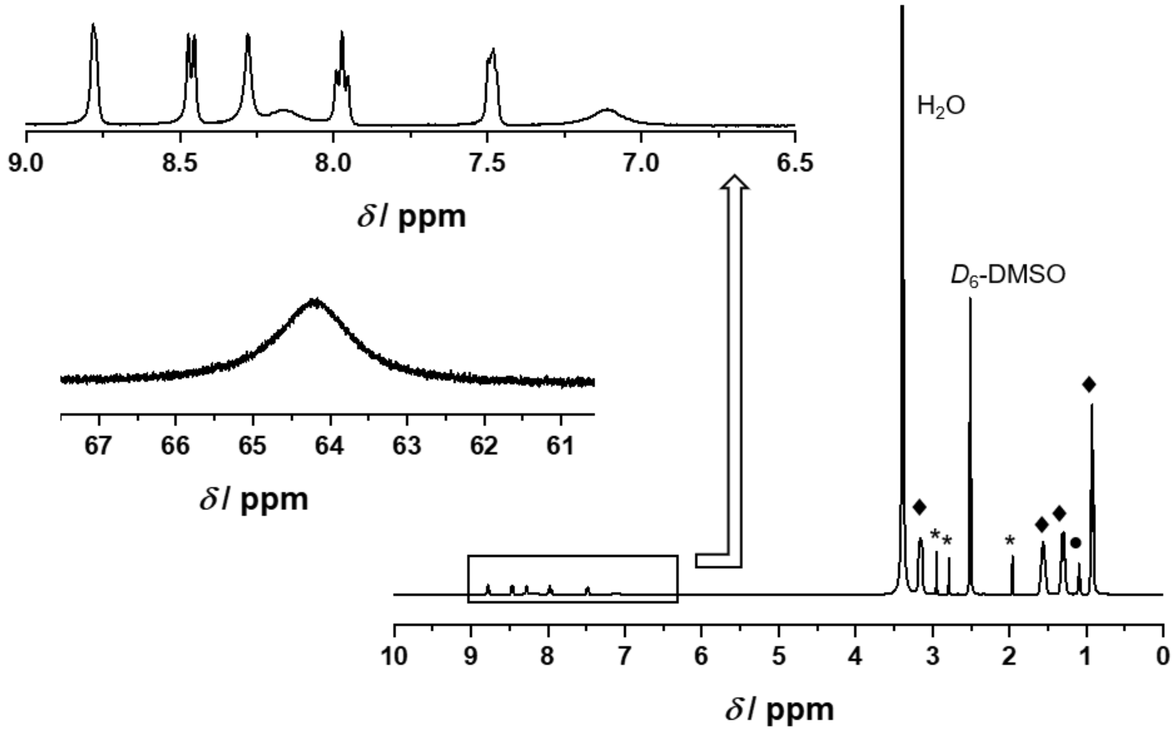

Figure SI- 9. ^{1}H NMR spectrum of **6** (400 MHz, D_6-DMSO). Residual DMAc (*), Et_2O (•) and TBA^+ signals (♦) are marked.

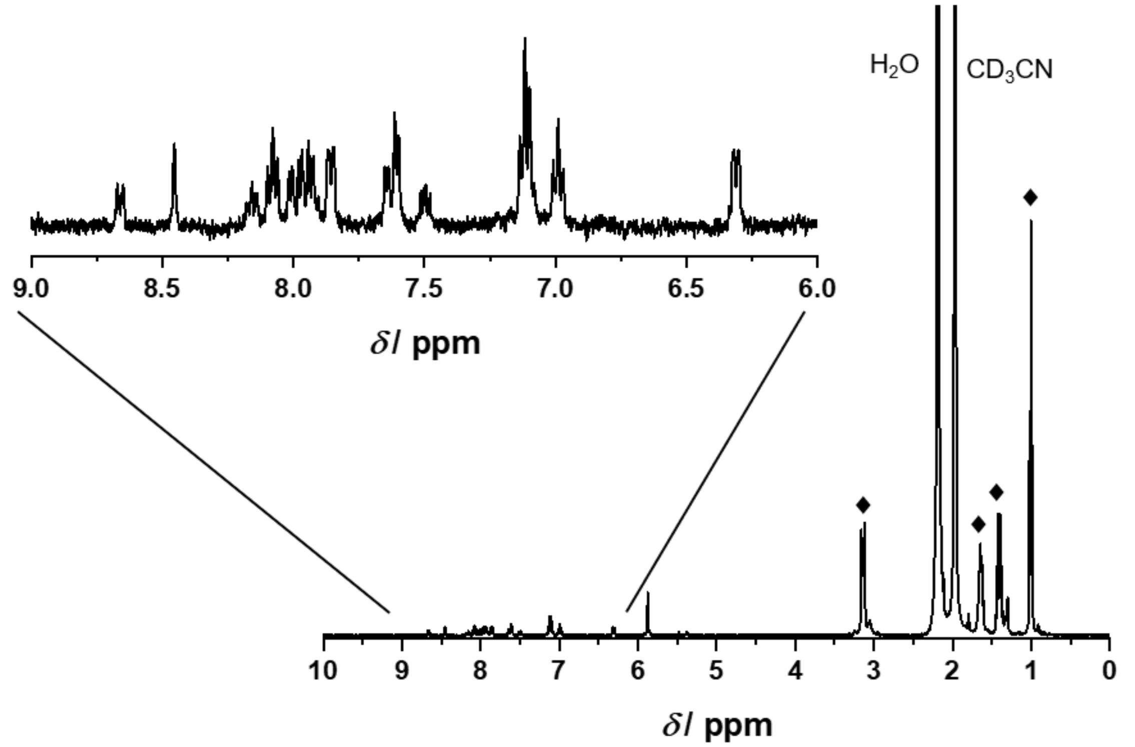

Figure SI- 10. ^{1}H NMR of **8** (400 MHz, CD_3CN). The TBA^+ signals (♦) are marked.

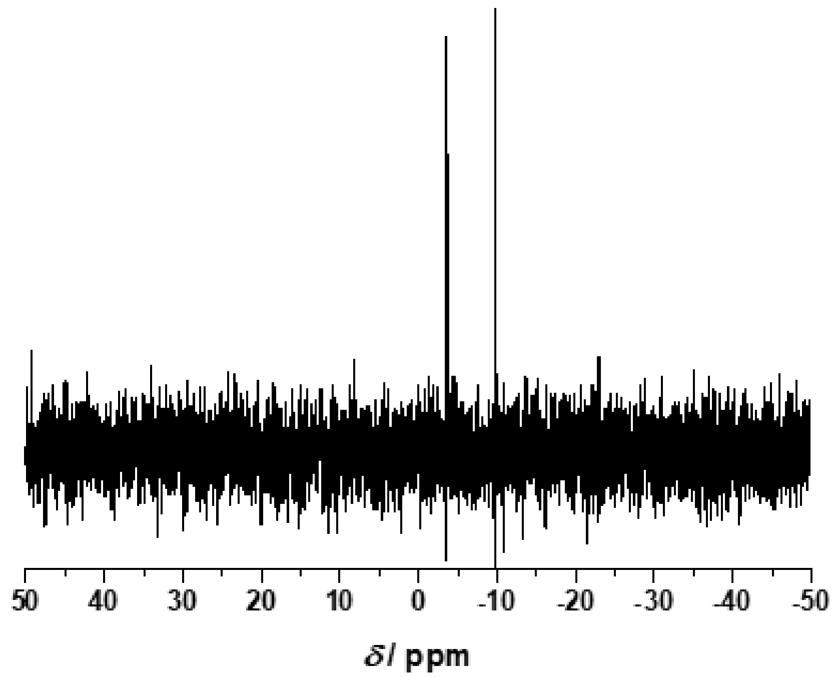

Figure SI- 11. ^{31}P NMR of **8** (162 MHz, CD_3CN).

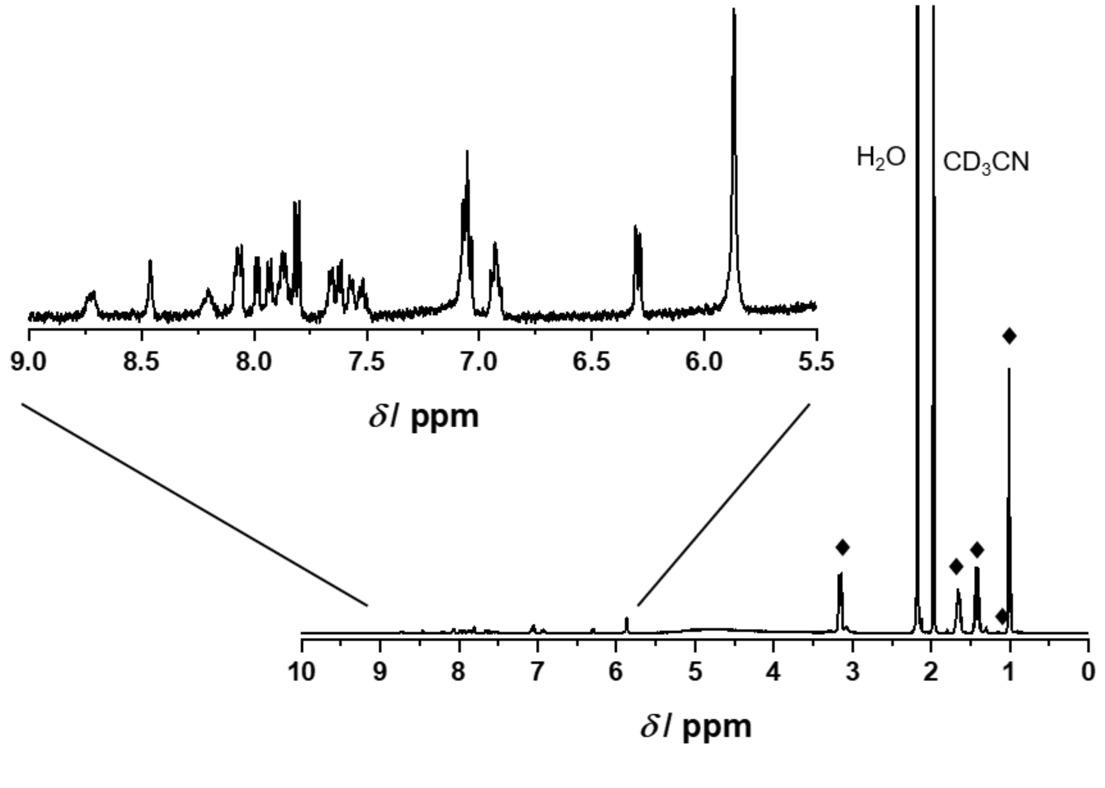

Figure SI- 12. ^{1}H NMR spectrum of **9** (400 MHz, CD_3CN). The TBA^+ signals (♦) are marked.

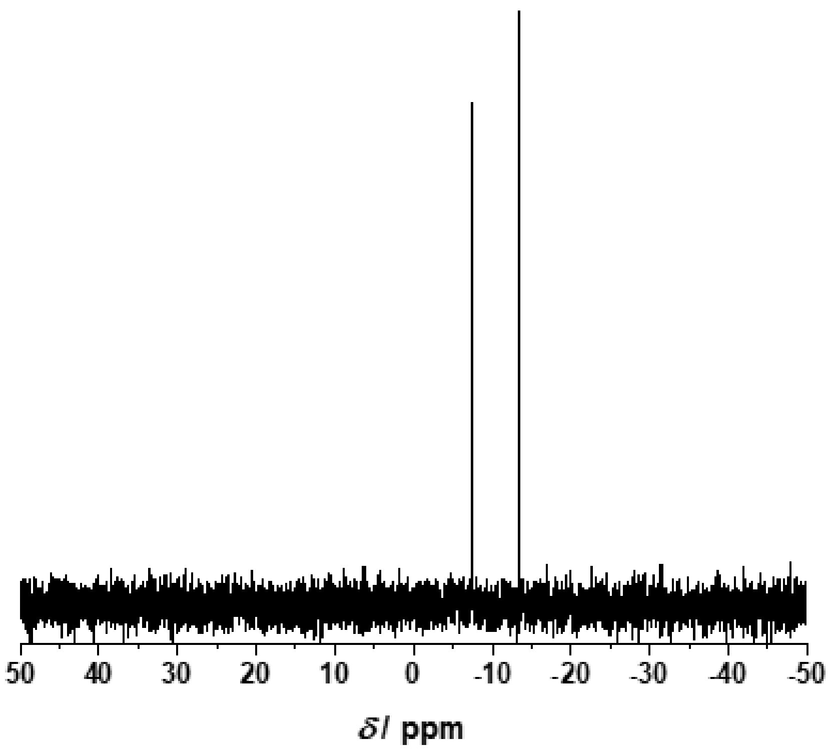

Figure SI- 13. ^{31}P NMR spectrum of **9** (162 MHz, CD_3CN).

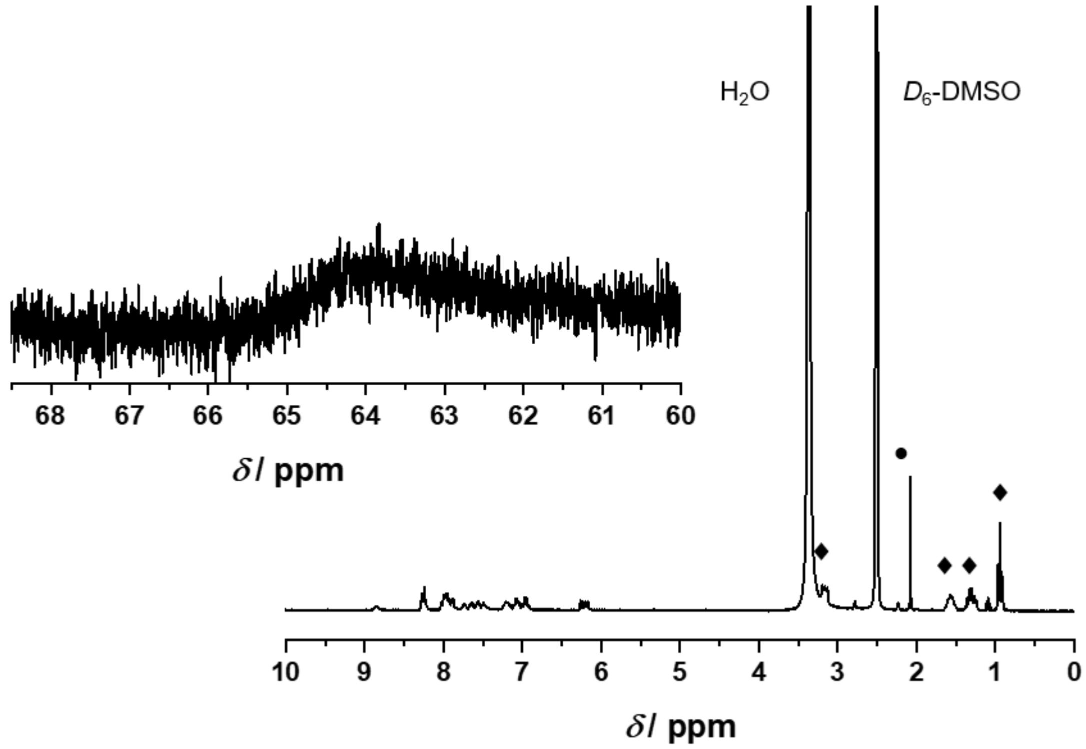

Figure SI- 14. ^{1}H NMR spectrum of **10** (400 MHz, *D*6-DMSO). Residual acetone (•) and TBA^+ signals (♦) are marked.

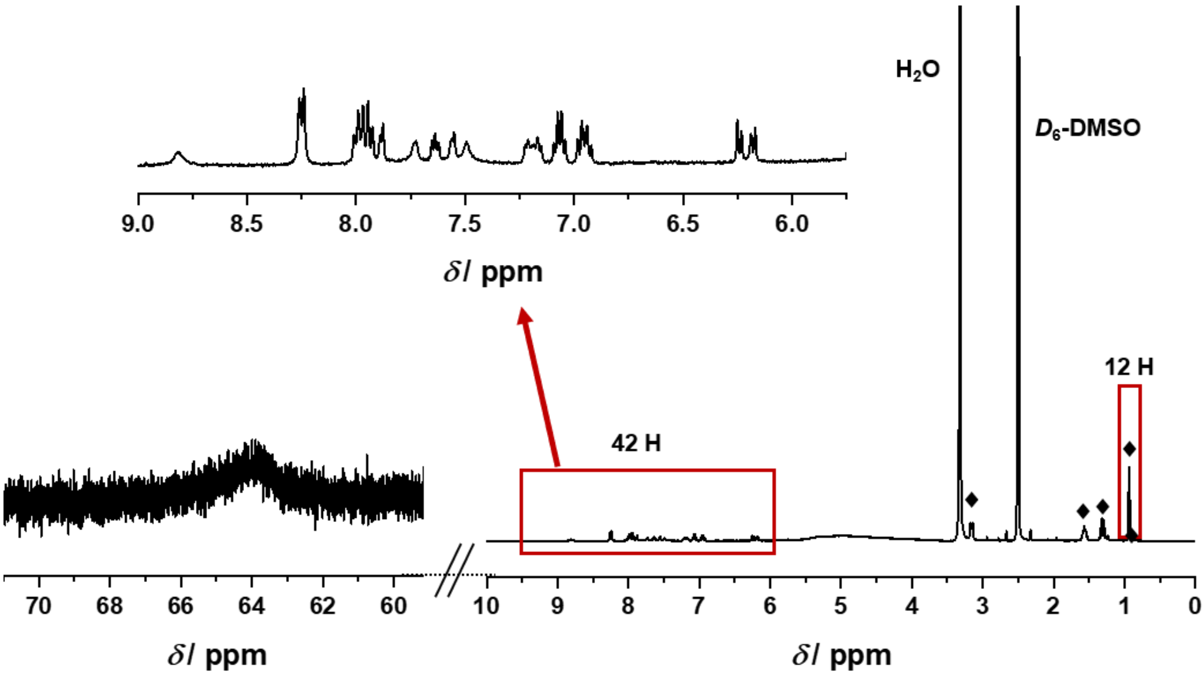

Figure SI- 15. ^{1}H NMR spectrum of **11** (400 MHz, *D*$_6$-DMSO). The TBA$^+$ signals (♦) are marked.

2. Mass spectrometry

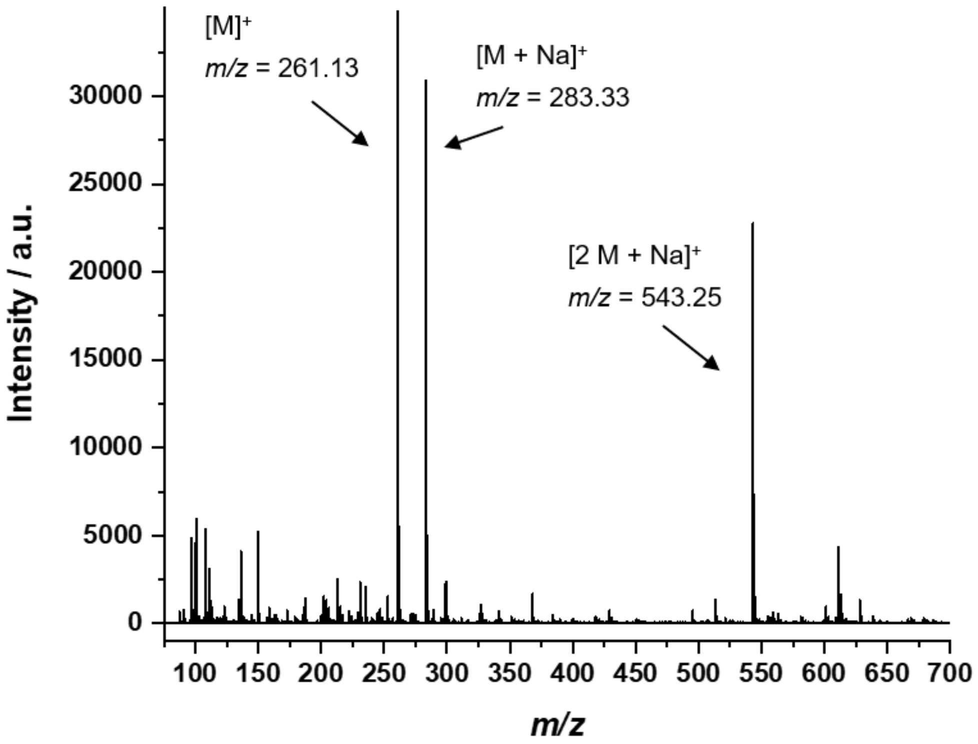

Figure SI- 16. ESI mass spectrum of **2** (positive mode).

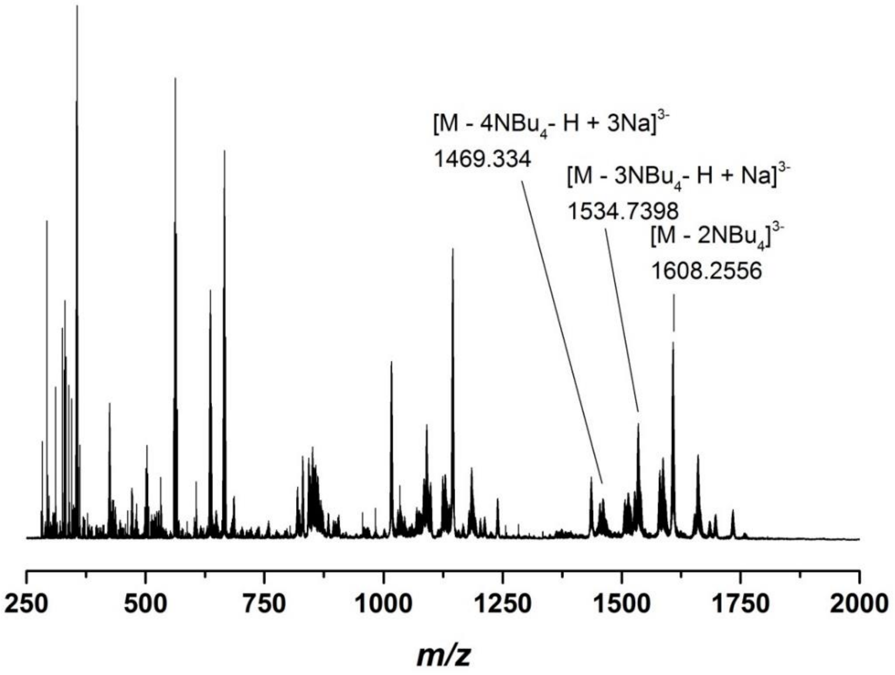

Figure SI- 17. ESI mass spectrum of **3** (negative mode).

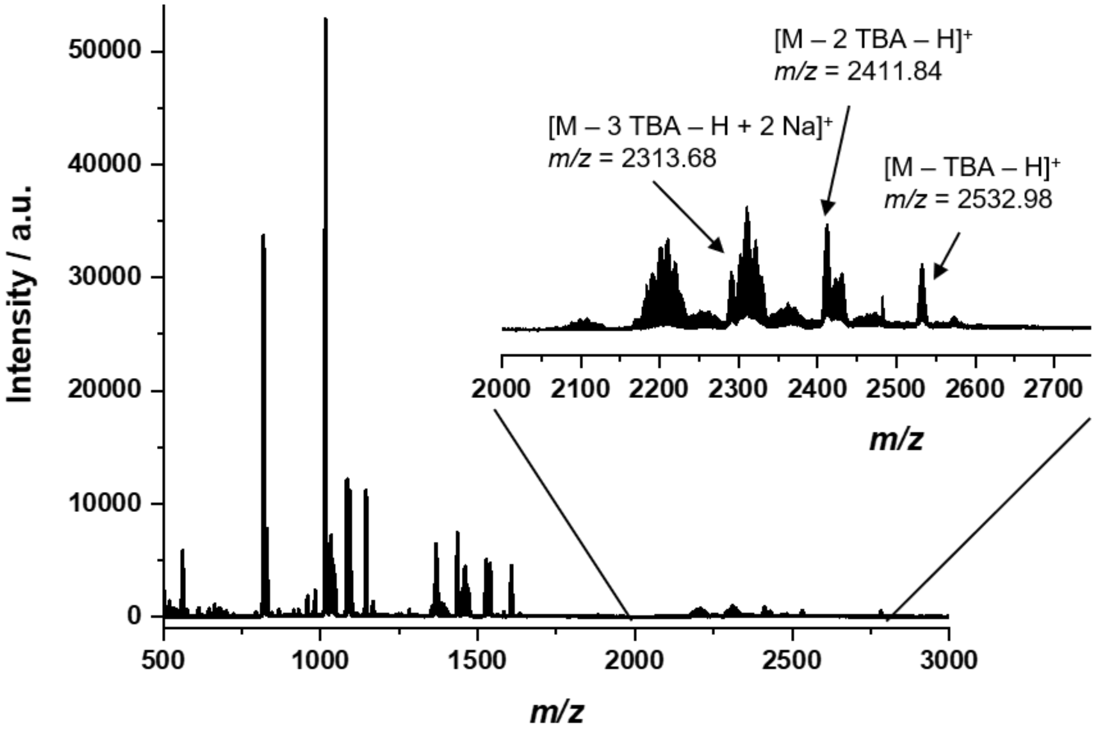

Figure SI- 18. MALDI-TOF mass spectrum of **3** (negative mode, DCTB as matrix, NaI as ionization salt).

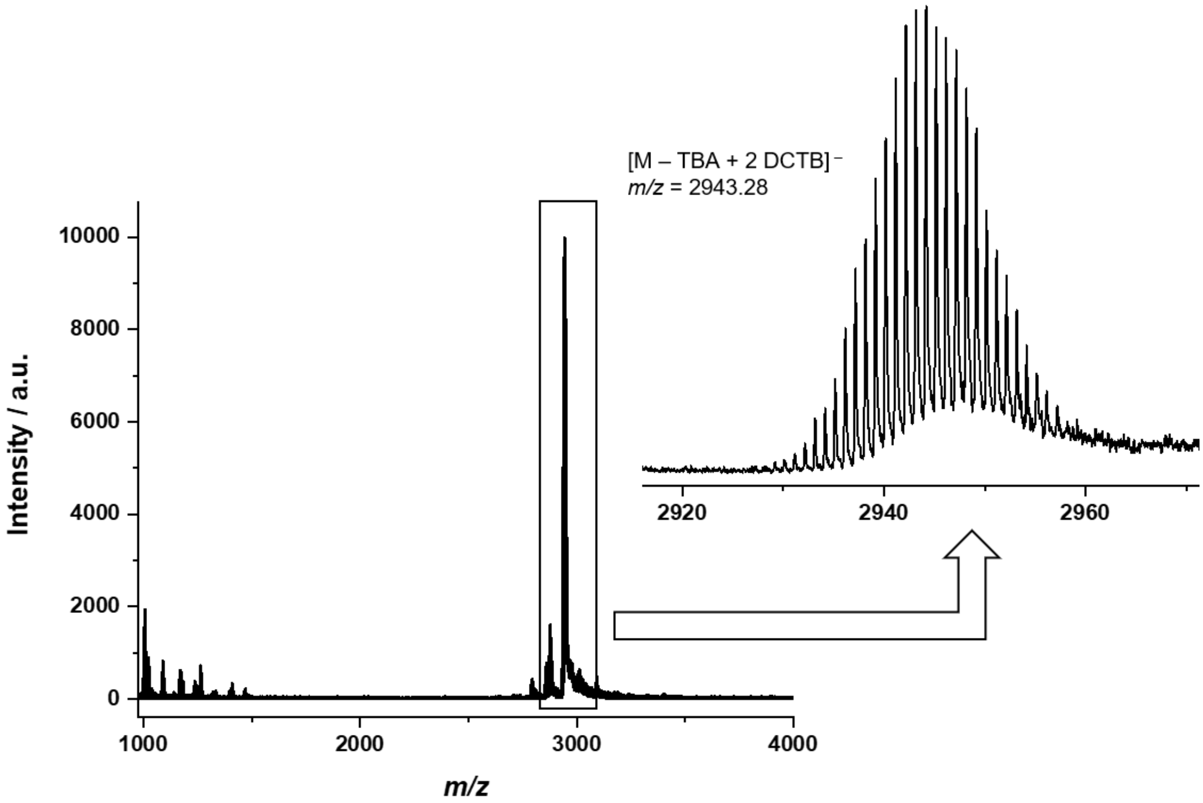

Figure SI- 19. MALDI-TOF mass spectrum of **4** (negative mode, DCTB as matrix, NaI as ionization salt).

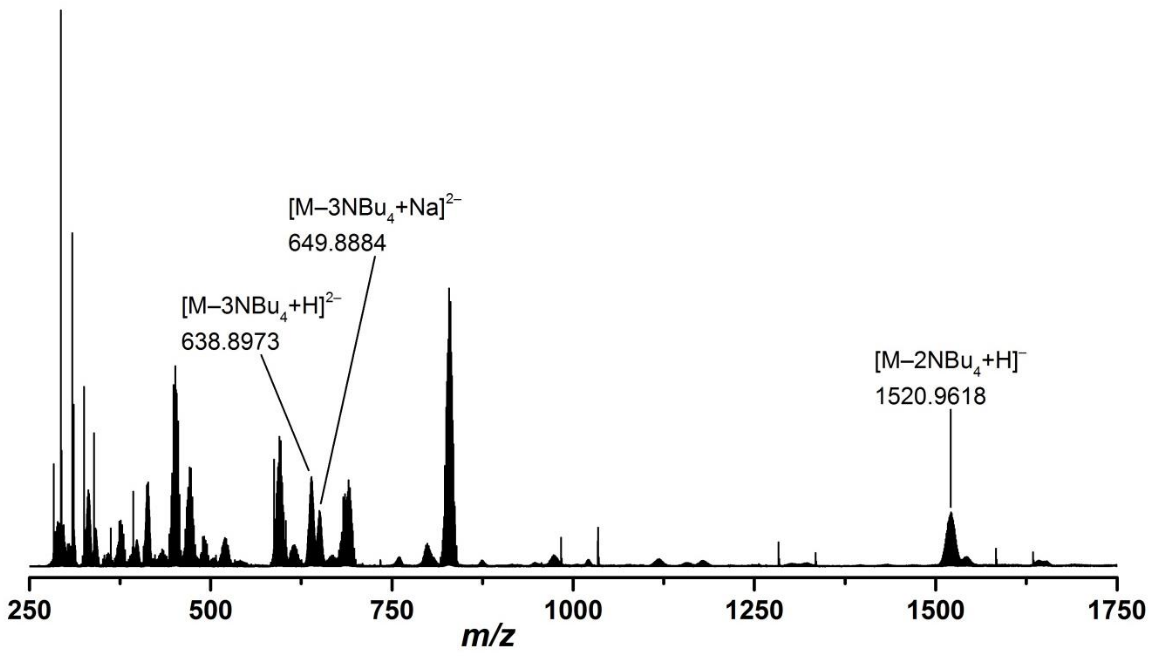

Figure SI- 20. ESI mass spectrum of **5** (negative mode).

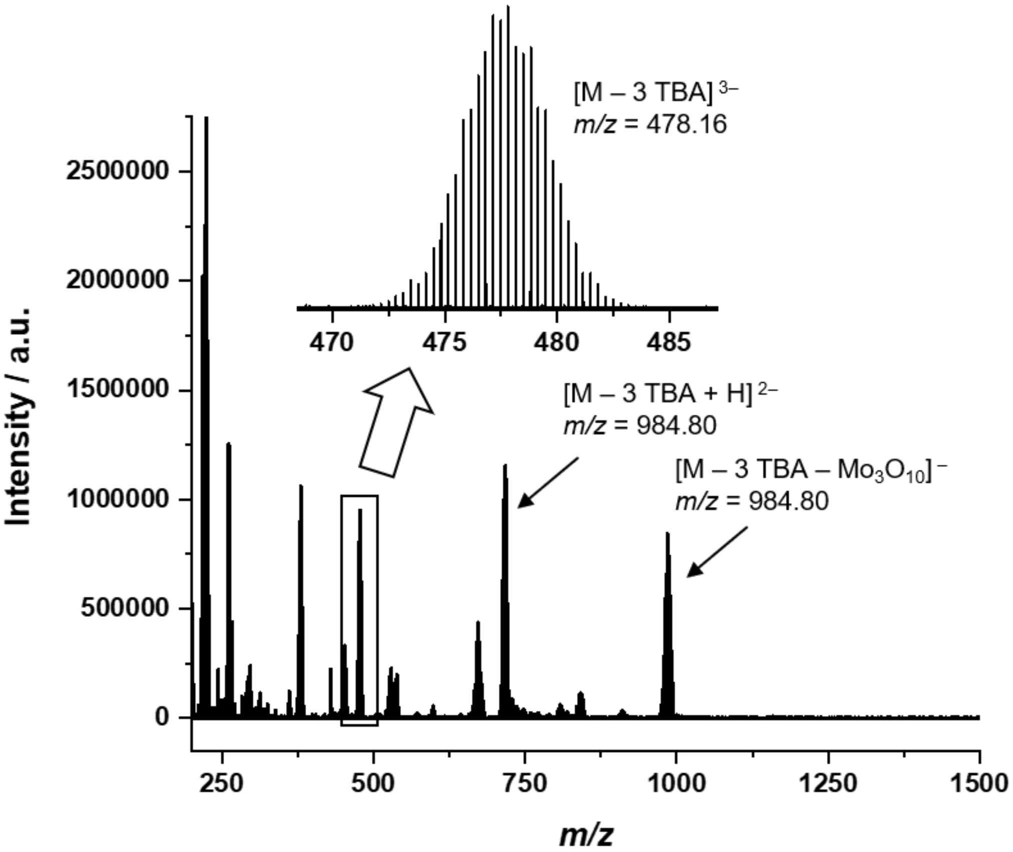

Figure SI- 21. ESI Mass spectrum of **6** (negative mode).

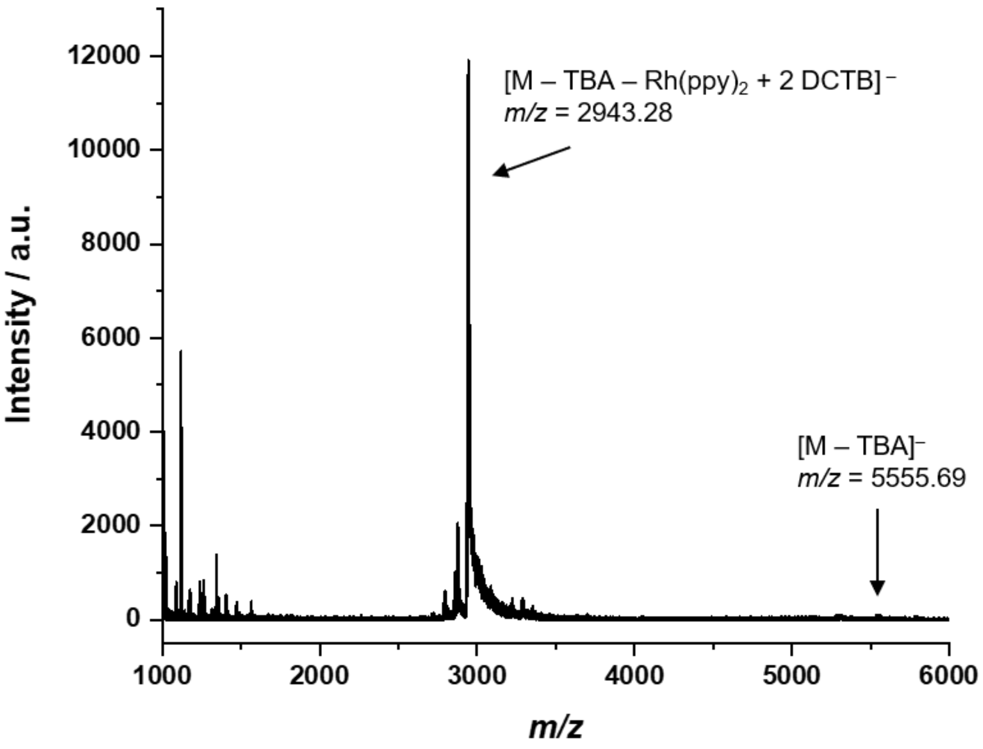

Figure SI- 22. MALDI-TOF mass spectrum of **8** (negative mode, DCTB as matrix, NaI as ionization salt).

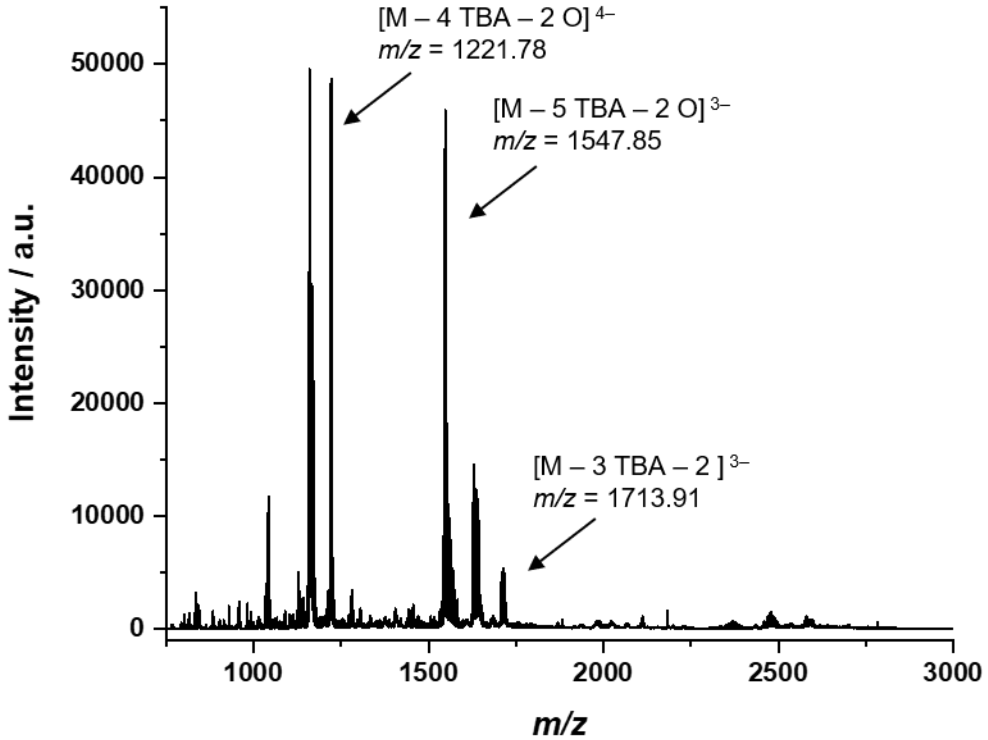

Figure SI- 23. ESI mass spectrum of **9** (negative mode).

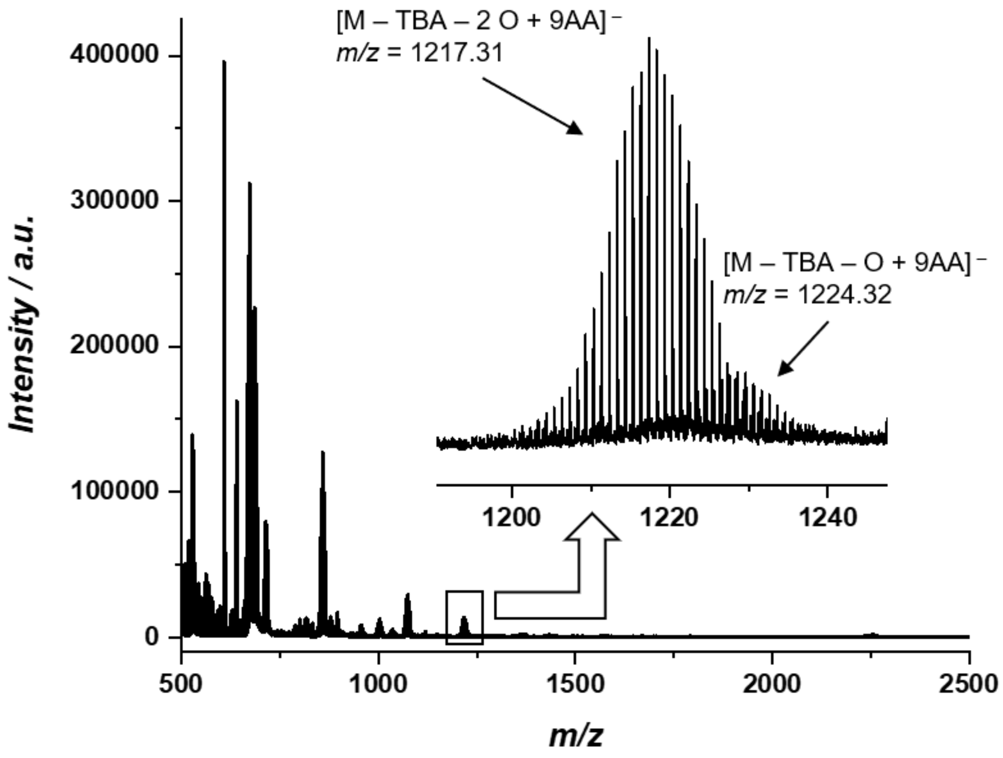

Figure SI- 24. MALDI-TOF mass spectrum of **10** (negative mode, 9-aminoacridine as matrix, NaI as ionization salt).

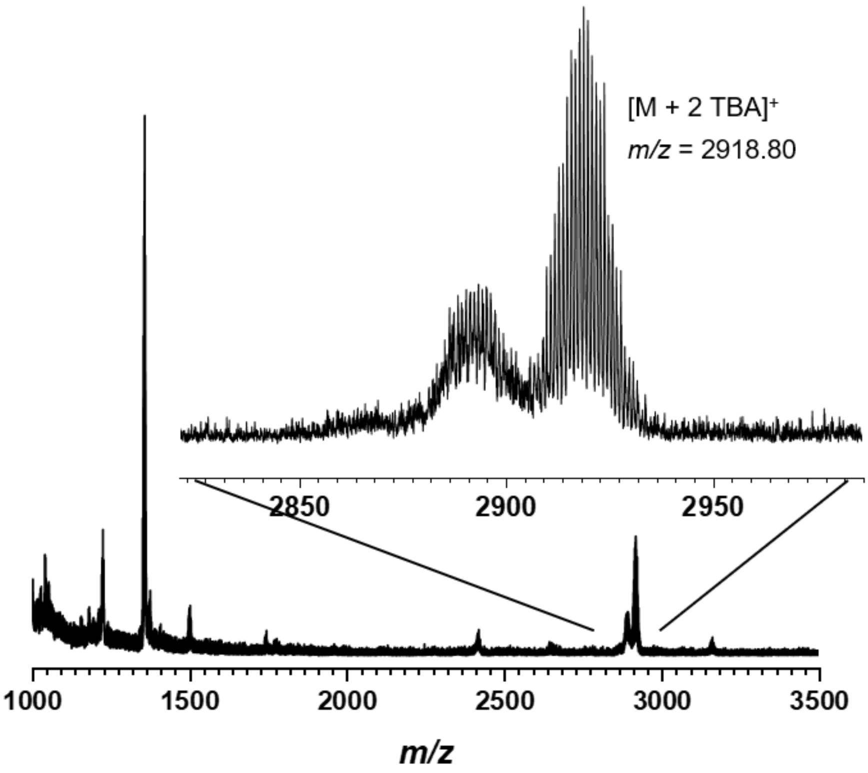

Figure SI- 25. MALDI-TOF mass spectrum of **11** (DCTB as matrix, KCl as ionization salt).

3. X-ray photoelectron spectroscopy

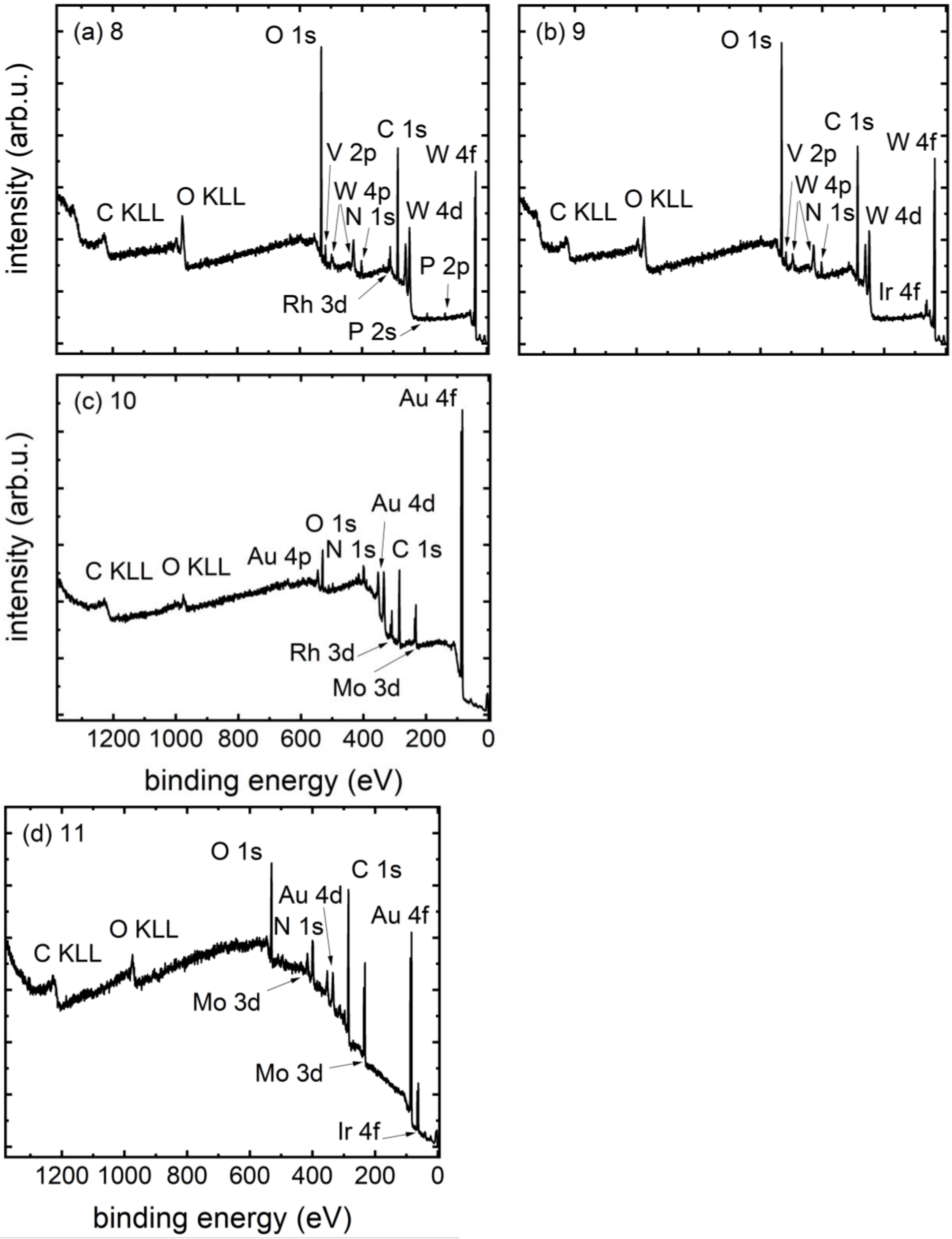

Figure SI- 26. XP overview spectra of the photosensitizer-POM dyads **8–11** with marked elements.

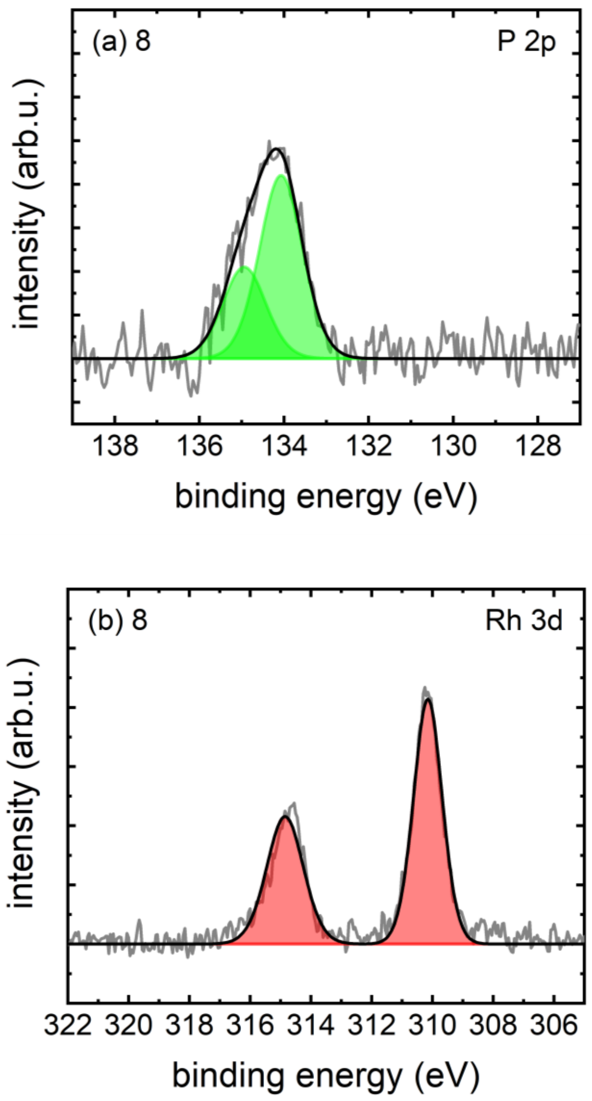

Figure SI- 27. High resolution XP P 2p and Rh 3d spectra of the photosensitizer-POM dyad **8**.

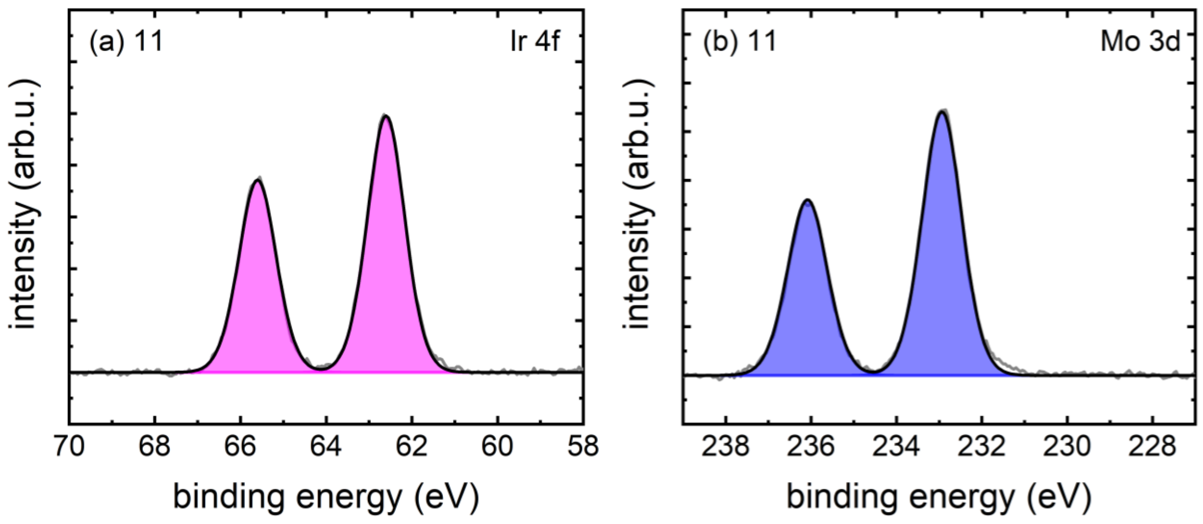

Figure SI- 28. High resolution XP Ir 4f and Mo 3d spectra of the photosensitizer-POM dyad **11**.

Table SI-1. Quantitative analysis of the high-resolution XP spectra of the photosensitizer-POM dyads **8–11** including peak assignment, binding energies and full width at half maximum (FWHM) values obtained from the spectra deconvolution.

Peak assignment	Binding energy, eV	FWHM, eV
photosensitizer-POM dyad **8**		
Rh $3d_{5/2}$		
$[(ppy)_2Rh]^+$	310.2	1.1
P $2p_{3/2}$		
$P_2W_{15}V_3O_{62}$	134.1	1.2
W $4f_{7/2}$		
$P_2W_{15}V_3O_{62}$	36.1	1.0
photosensitizer-POM dyad **9**		
Ir $4f_{7/2}$		
$[(ppy)_2Ir]^+$	63.0	1.2
P $2p_{3/2}$		
$P_2W_{15}V_3O_{62}$	134.3	1.2
W $4f_{7/2}$		
$P_2W_{15}V_3O_{62}$	36.4	1.0
photosensitizer-POM dyad **10**		
Rh $3d_{5/2}$		
$[(ppy)_2Rh]^+$	309.8	1.0
Mo $3d_{5/2}$		
$MnMo_6O_{24}$	232.8	1.1
Mn $2p_{3/2}$		
$MnMo_6O_{24}$	642.5	5.0
photosensitizer-POM dyad **11**		
Ir $4f_{7/2}$		
$[(ppy)_2Ir]^+$	62.6	1.0
Mo $3d_{5/2}$		
$MnMo_6O_{24}$	232.9	1.1

Mn $2p_{3/2}$		
$MnMo_6O_{24}$	642.8	4.8

The peak fitting of the doublets was performed using fixed intensity ratios due to the spin-orbit coupling of the p, d and f photoelectrons, respectively. With respect to the determination of the elemental ratio (see the main manuscript), the following relative sensitivity factors (RSF) were used: 8.39 (Rh $3d_{5/2}$), 0.79 (P $2p_{3/2}$), 7.78 (Ir $4f_{7/2}$), 5.62 (Mo $3d_{5/2}$), 9.17 (Mn $2p_{3/2}$) and 5.48 (W $4f_{7/2}$).

4. Cyclic and square-wave voltammetry

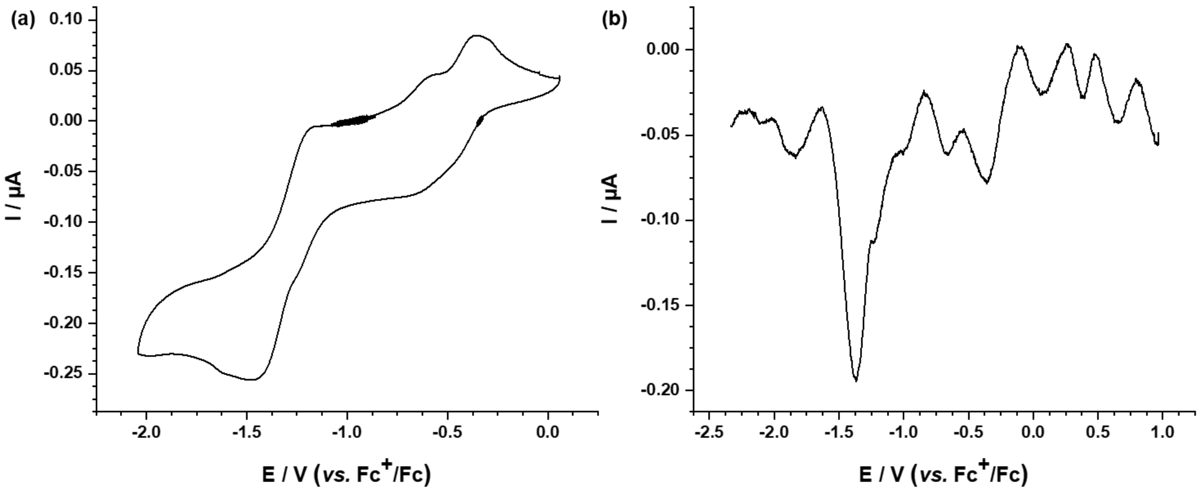

Figure SI- 29. Cyclic (a) and square-wave voltammograms (b) of **3**. CV and SWV were measured at room temperature in degassed CH_3CN containing 0.1 M $(TBA)PF_6$ (scan rate of 100 mV/s).

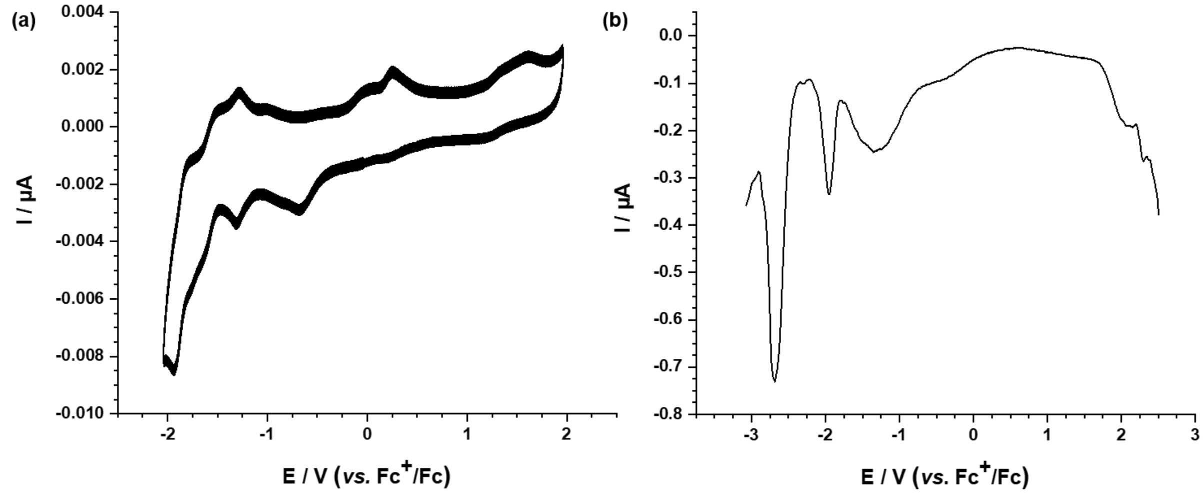

Figure SI- 30. Cyclic (a) and square-wave voltammograms (b) of **8**. CV and SWV were measured at room temperature in degassed CH_3CN containing 0.1 M $(TBA)PF_6$ (scan rate of 200 mV/s).

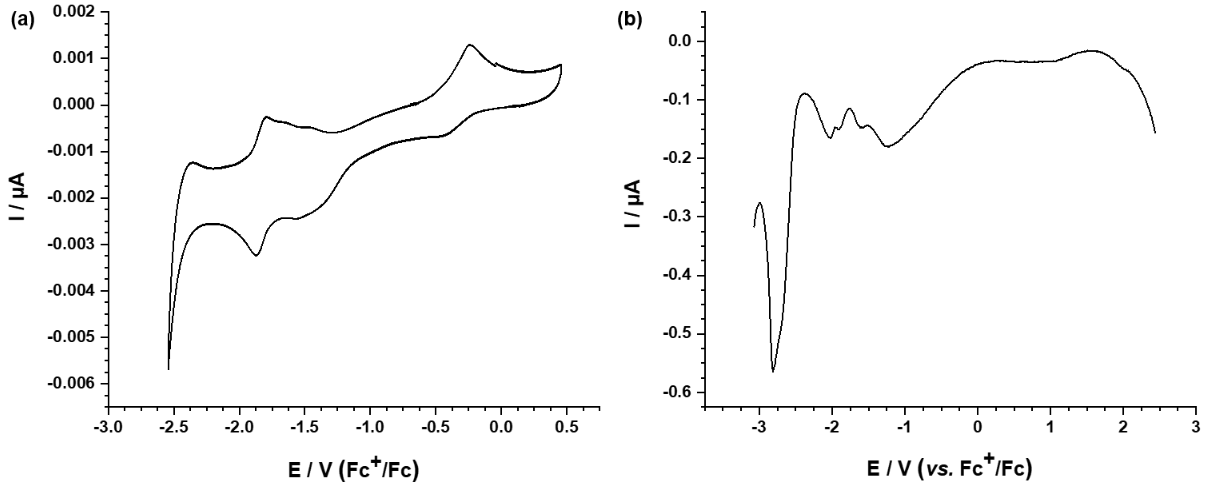

Figure SI- 31. Cyclic (a) and square-wave voltammograms (b) of **9**. CV and SWV were measured at room temperature in degassed CH_3CN containing 0.1 M $(TBA)PF_6$ (scan rate of 200 mV/s).

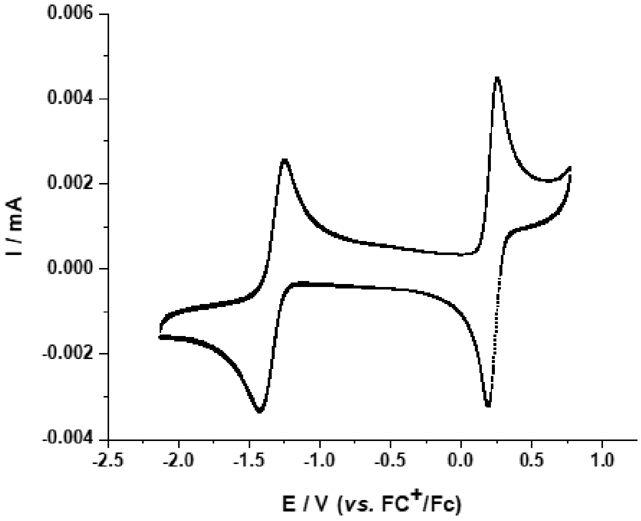

Figure SI- 32. Cyclic voltammogram of **5** measured at room temperature in degassed DMF containing 0.1 M $(TBA)PF_6$ (scan rates of 100 mV/s).

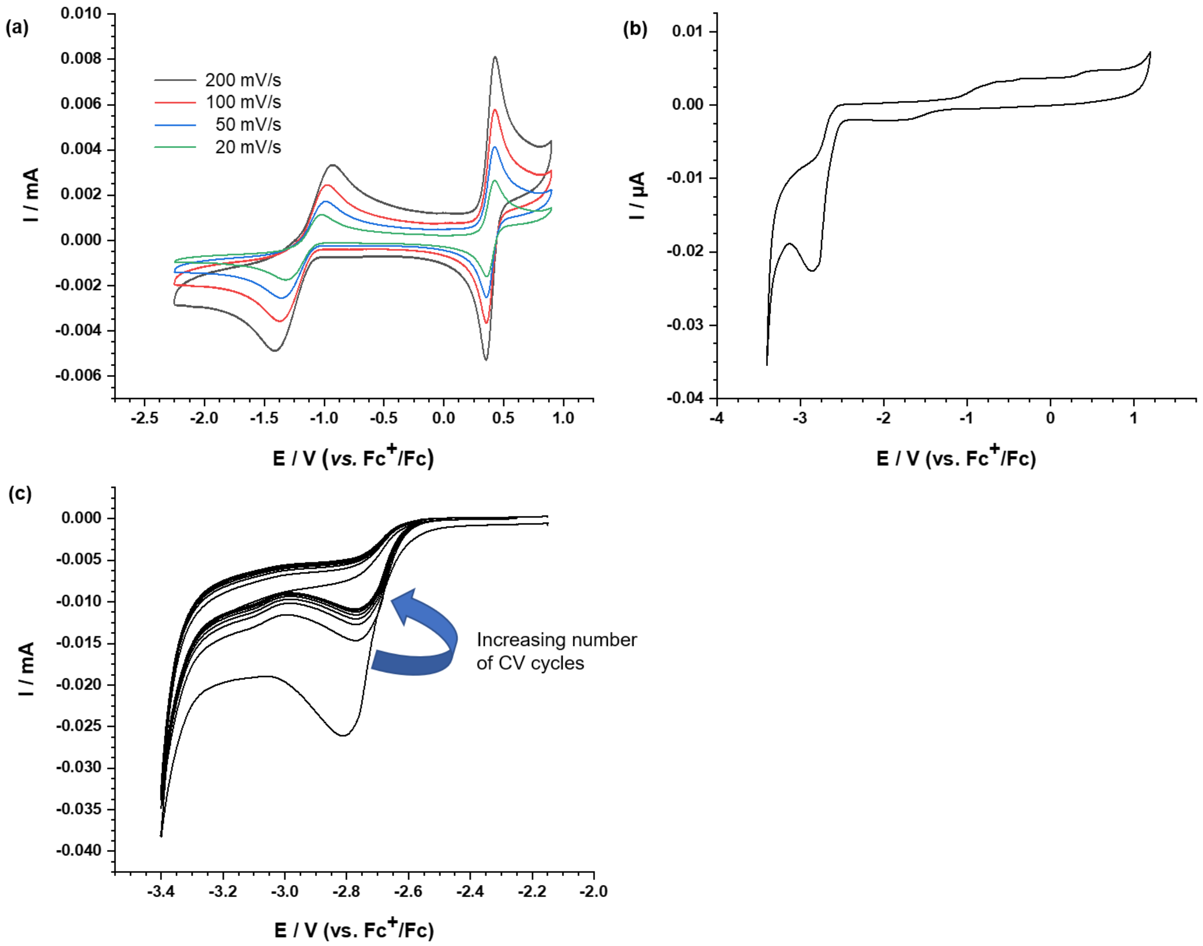

Figure SI- 33. Cyclic (a and c) and square-wave voltammograms (b) of **6**. CV and SWV were measured at room temperature in degassed CH_3CN containing 0.1 M $(TBA)PF_6$. Different scan rates were used in the CV measurements in the potential range from –2.25 to 1 V.

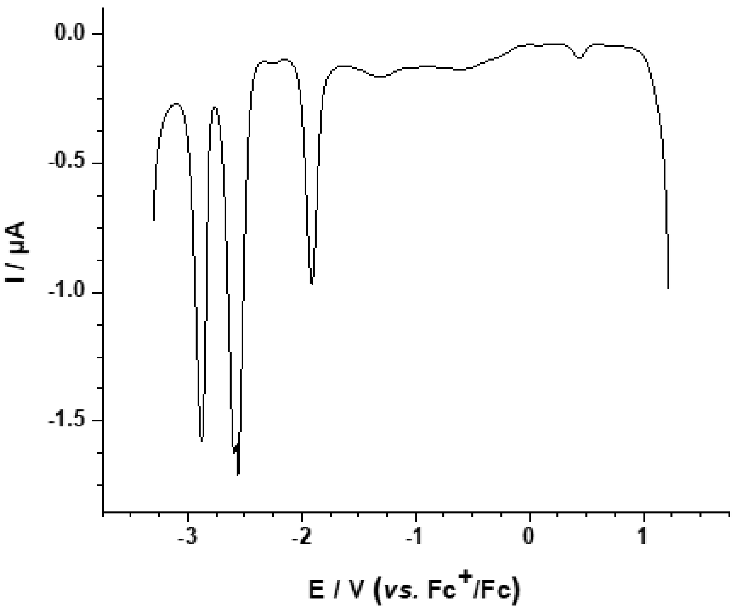

Figure SI- 34. Square-wave voltammogram of **10** measured at room temperature in degassed DMF containing 0.1 M $TBPF_6$ (scan rates of 100 mV/s).

5. UV/vis absorption and emission spectroscopy

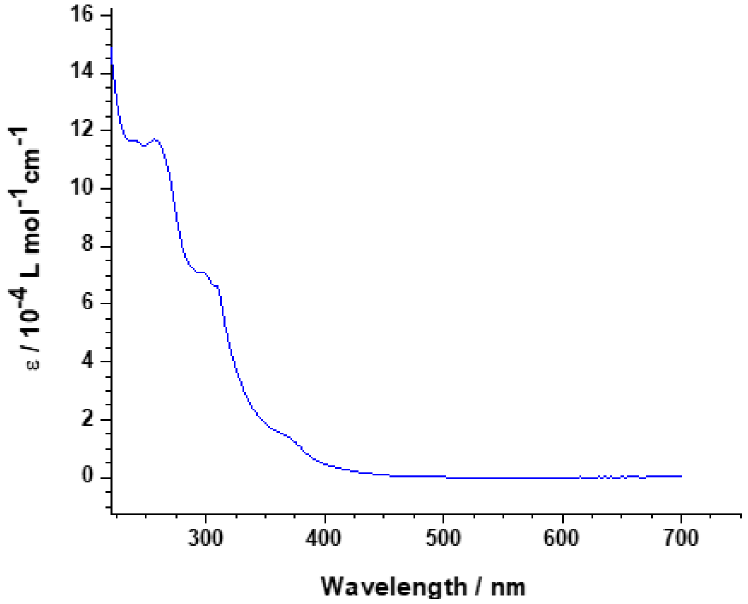

Figure SI- 35. UV/vis absorption spectrum of the Rh(III)-containing dyad **8**.
